# CCT2 Orchestrates Glycolysis and Exosome-Mediated M2 Macrophage Polarization in HCC tumorigenesis

**DOI:** 10.7150/ijbs.122859

**Published:** 2026-03-17

**Authors:** Wing-Wa Guo, Cairang Dongzhi, Tianyin Ma, Minghe Zhang, Meng Gao, Xiaomian Li, Jitong Zhou, Xi Chen, Wenzhi He, Ming Tian, Yufeng Yuan, Weijie Ma

**Affiliations:** 1 Department of Hepatobiliary & Pancreatic Surgery, Zhongnan Hospital of Wuhan University, Wuhan, China.; 2 Clinical Medicine Research Center for Minimally Invasive Procedure of Hepatobiliary & Pancreatic Diseases of Hubei Province, Hubei, PR China.; 3 Taikang Center for Life and Medical Sciences of Wuhan University, Wuhan, China.

**Keywords:** CCT2, exosome, hepatocellular carcinoma, macrophage, glycolysis

## Abstract

**Introduction:**

Hepatocellular carcinoma (HCC) is a highly aggressive malignancy with a poor prognosis, driven by metabolic reprogramming and immune evasion. The role of T-complex protein 1 subunit beta (CCT2) in HCC remains unclear. This study aimed to elucidate the function of CCT2 in HCC tumorigenesis.

**Methods:**

Bioinformatics analysis and Clinical samples investigation were integrated with *in vitro* and *in vivo* experiments to investigate CCT2's role in HCC metabolism and immune modulation. The glycolytic activity was assessed by measuring extracellular acidification rate, glucose uptake, lactate levels, and metabolomic profiles. Coimmunoprecipitation or GST pulldown assays confirmed CCT2 interactions with aldolase A (ALDOA) and glutathione S-transferase P (GSTP1), while THP1 co-culture assays evaluated tumor immune crosstalk.

**Results:**

CCT2 directly interacts with and stabilizes the glycolytic enzyme ALDOA, as shown by co-immunoprecipitation and metabolic assays revealing increased extracellular acidification rate, glucose uptake, and lactate production in HCC cells. Genetic depletion of CCT2 suppresses tumor cell proliferation and migration *in vitro* and inhibits tumor growth *in vivo*. Furthermore, co-culture and exosome treatment experiments reveal that CCT2 promotes M2 macrophage polarization and establishes an immunosuppressive tumor microenvironment through coordinated metabolic and exosome-mediated mechanisms. In mouse models, CCT2 knockdown significantly enhances the antitumor efficacy of PD-1 blockade.

**Conclusions:**

CCT2 stabilizes ALDOA and facilitates exosome-mediated immunosuppressive signaling, thereby linking metabolic reprogramming to immune evasion in HCC and supporting its potential as a mechanistically informed therapeutic target.

## Introduction

Hepatocellular carcinoma (HCC) is among the most prevalent cancers worldwide, ranking sixth in incidence and third in cancer-related mortality 1,2. Due to its asymptomatic progression, most cases are diagnosed at advanced stages 3,4. Despite advances in surgery, targeted therapies, and immunotherapy, the 5-year overall survival rate for advanced HCC remains below 15% 5,6. The limited understanding of the cellular mechanisms driving HCC development and its associated intercellular communication compromises the effectiveness of current treatment strategies, leading to poor patient outcomes 7,8. Thus, further investigation is urgently needed to identify novel therapeutic targets for more effective treatments.

The pathogenesis of HCC is influenced by a multifaceted tumor microenvironment (TME), which contributes to rapid disease progression 8,9. Within the TME, cancer cells demonstrate remarkable adaptability, employing various mechanisms to facilitate their development and progression 10. Tumor cells secrete cytokines and exosomes that remodel the TME, enhancing angiogenesis, recruiting immune-suppressive cells, and promoting immune tolerance 11,12. Moreover, metabolic reprogramming can also alter TME 13,14, with glycolysis playing a pivotal role in HCC 15. HCC cells often exhibit increased aerobic glycolysis, a hallmark of the Warburg effect 16,17. This metabolic shift supports rapid cell proliferation by generating ATP and providing essential intermediates for biosynthesis 18. Furthermore, glycolysis produces lactate, which acidifies the TME, impairs immune cell function, and triggers immune evasion 19,20.

Notably, lactate influences tumor-associated macrophages (TAMs), particularly M2-polarized macrophages, which foster a pro-tumorigenic environment by promoting angiogenesis, metastasis, and immune suppression 21,22. Lactate-induced M2 polarization is mediated by macrophage activation and increased secretion of immunosuppressive cytokines, such as IL-10 and TGF-β 23. However, the precise molecular mechanisms by which glycolysis in HCC cells regulate M2 macrophage polarization remain poorly understood.

T-complex protein 1 subunit beta (CCT2), a component of the chaperonin-containing TCP1 complex (TRiC/CCT), functions as a molecular chaperone involved in folding key proteins, including tubulin 24. Previous studies have demonstrated that CCT2 contributes to tumorigenesis in several cancer types 25,26. However, its functional role in HCC progression and the underlying molecular mechanisms remain to be elucidated. In this study, we demonstrate that CCT2 promotes glycolytic activity in HCC cells by stabilizing the key glycolytic enzyme aldolase A (ALDOA), thereby enhancing lactate production and facilitating lactate-dependent M2 macrophage polarization. These findings identify CCT2 as a regulator that links metabolic reprogramming to immune modulation in HCC.

Intriguingly, inhibition of lactate production only partially attenuated the immunosuppressive effects induced by CCT2-overexpressing HCC cells, suggesting the involvement of additional tumor-macrophage communication mechanisms beyond lactate signaling. Given the central role of exosomes in intercellular communication within the tumor microenvironment 27,28, we investigated whether CCT2 participates in exosome-mediated immune regulation. Our results indicate that CCT2 promotes M2 macrophage polarization through both metabolic and exosome-dependent pathways, thereby establishing an immunosuppressive microenvironment that supports HCC progression, and highlighting CCT2 as a multifunctional therapeutic target.

## Materials and Methods

### Human samples

The clinical samples used in this study were obtained from 23 patients who underwent curative resection for HCC at Zhongnan Hospital of Wuhan University (Hubei Province, China). Written informed consent was obtained from all participants prior to their sample collection. Paired HCC tumor tissues and adjacent non-tumor liver tissues were collected, and their relevant clinical information was obtained. No formal sample size calculation was performed, as the number of tissue samples was determined based on their availability. Patients were selected using a simple random-sampling method to minimize any potential selection bias. The inclusion criteria required a confirmed diagnosis of primary HCC by two independent pathologists and eligibility for surgical resection without prior exposure to chemotherapy or radiotherapy. Patients with a history of preoperative chemotherapy or radiotherapy, or those diagnosed with non-primary liver malignancies, were excluded. This study was conducted in accordance with the principles of the Declaration of Helsinki and was approved by the Ethics Committee of Zhongnan Hospital of Wuhan University (Approval No. 2022181K).

### Murine xenograft assay

Five-week-old male BALB/c nude mice were obtained from Wuhan University Laboratory Animal Center. HCCLM3 cells stably transfected with shNC and shCCT2 were subcutaneously injected into the right axilla of the mice. Similarly, Hep3B cells stably transfected with oeNC and oeCCT2 were used for the subcutaneous tumor implantation in BALB/c nude mice. In addition, C57BL/6 mice were injected with Hep53.4 cells stably transfected with shNC and shCCT2 for the subcutaneous tumor experiment. The tumors were collected after 25 days. In the C57BL/6 mouse experiment, anti-programmed cell death protein 1 monoclonal antibody (anti-PD-1 mAb) (10 mg/kg, i.p.) was administered as a part of the treatment regimen. For the orthotopic HCC model, shNC- or shCCT2-transduced HCC cells were orthotopically implanted into the liver by intrahepatic injection, followed by treatment with phosphate-buffered saline (PBS) or anti-PD-1 mAb. Mice were divided into four groups (shNC+PBS, shcct2+PBS, shNC+PD-1 mAb, and shcct2+PD-1 mAb). All animal experimental procedures were reviewed and approved by the Institutional Animal Care and Use Committee (IACUC) of Wuhan University Animal Experimentation Center and were conducted in accordance with the Guidelines for the Care and Use of Laboratory Animals of the Chinese Animal Welfare Committee.

### Cell culture

Human hepatocellular carcinoma cell lines including Hep3B (RRID: CVCL_0326), Li-7 (RRID: CVCL_3840), Huh7 (RRID: CVCL_0336), PLC/PRF/5 (RRID: CVCL_0485), and HCCLM3 (RRID: CVCL_6832), as well as the monocytic cell line THP1 (RRID: CVCL_0006), were obtained from the Cell Bank of the Chinese Academy of Sciences (Shanghai, China). The normal human liver cell line THLE-2 (RRID: CVCL_3803) and the human embryonic kidney cell line HEK293T (RRID: CVCL_0063) were purchased from ATCC (Manassas, VA, USA). The Hep53.4 cell line (RRID: CVCL_5765) was purchased from IMMOCELL (Xiamen, China).

THLE-2 cells were cultured in bronchial epithelial cell basal medium supplemented with the BEGM Bullet Kit (CC-3170, Lonza). Huh7, PLC/PRF/5, HCCLM3, HEK293T, and Hep53.4 cells were cultured in DMEM (Thermo Fisher Scientific) supplemented with 10% fetal bovine serum (FBS; Gibco), with Hep53.4 additionally supplemented with 1.5 g/L NaHCO₃. Hep3B cells were maintained in MEM medium supplemented with 10% FBS, 1% non-essential amino acids, and 1% sodium pyruvate. Li-7 and THP1 cells were cultured in RPMI-1640 medium with 10% FBS. THP1 cells were differentiated into macrophage-like cells by treatment with 150 ng/mL phorbol-12-myristate-13-acetate (PMA; MedChemExpress) for 48 hours.

All cell lines were cultured at 37 °C in a humidified atmosphere containing 5% CO₂. Mycoplasma contamination was routinely tested and ruled out using polymerase chain reaction-based detection kits. Short Tandem Repeat (STR) profiling was performed on key cell lines for authentication. HEK293T cells, derived from human embryonic kidney cells and expressing SV40 large T antigen, were used for lentiviral production and other plasmid-based transfection assays. The use of multiple cell lines and validation across different experimental systems ensure the robustness and reproducibility of the findings.

For co-culture experiments, indicated HCC cells were seeded into the upper chamber of a Transwell insert (0.4 μm pore size, Millipore, USA). For exosome treatment, isolated exosomes (1 μg/mL) were added directly to the culture medium of recipient cells.

### Generation of stable cell lines

Stable cell lines were generated from Huh7, HCCLM3, and Hep3B human hepatocellular carcinoma cell lines. In Huh7 cells, CCT2 knockdown was achieved using shRNA constructs (shCCT2-1, shCCT2-2) with PLKO.1 vectors, followed by puromycin selection. CCT2 overexpression was induced with a Flag-tagged CCT2 construct using the pLV3-EF1a-MCS-3×FLAG-Puro vector. ALDOA knockdown was performed with shRNA targeting ALDOA, and co-transfections of Flag-CCT2 with shALDOA or Flag-CCT2 with shGSTP1 were conducted for stable co-expression. HA-tagged ALDOA and GSTP1 constructs were overexpressed using pLV3-EF1a-MCS-3×HA-Puro, and stable clones were selected with puromycin. Furthermore, combinations of shCCT2-1 with HA-ALDOA, shCCT2-1 with HA-GSTP1, and shNC with HA-ALDOA were co-transfected to study the effects of these protein interactions. Co-transfections of shCCT2-1 with HA-GSTP1 and shNC with HA-GSTP1 were also performed, followed by puromycin selection for stable clones. For the HCCLM3 cell line, shCCT2-1 and shCCT2-2 constructs were transfected for CCT2 knockdown, with shNC as a control. In Hep3B cells, Flag-CCT2 overexpression was achieved using the Flag-CCT2 construct, with oeNC as the control. For the Hep53.4 cell line, shcct2-1 and shcct2-2 constructs were transfected for CCT2 knockdown, with shNC as a control. Control cell lines were generated using non-targeting shRNA (shNC) and an empty vector (oeNC) as appropriate.

### Detection of the targeted metabolites

All metabolites were detected by MetWare (http://www.metware.cn/) based on the AB Sciex QTRAP 6500 LC-MS/MS platform.

### Cellular metabolism assessment

Glycolytic function was assessed using the Seahorse XF Glycolysis Stress Test Kit (Agilent Technologies) according to the manufacturer's instructions. Briefly, cells were incubated in XF Base Medium supplemented with 2 mM glutamine, without glucose or pyruvate, with pH adjusted to 7.4 at 37 °C, and equilibrated for 1 h in a non-CO₂ incubator prior to measurement. Extracellular acidification rate (ECAR) was recorded under basal conditions followed by sequential injections of glucose (final 10 mM), oligomycin (final 1 μM), and 2-deoxy-D-glucose (2-DG, final 50 mM) using a 3-min mix, 2-min wait, and 3-min measure cycle throughout the assay. After completion of the Seahorse assay, cells were stained with Hoechst 33342, and fluorescence images were acquired using an imaging microplate reader. Cell numbers were determined by automated counting of Hoechst-positive nuclei and used to normalize ECAR values.

### Glucose uptake assay

Cells were incubated with 50 μM 2-NBDG (2-Deoxy-2-[(7-nitro-2,1,3-benzoxadiazol-4-yl) amino]-d-glucose; Topscience) in glucose-free culture medium at 37°C for 30 minutes, followed by washing with PBS and resuspension in fluorescence-activated cell sorting (FACS) buffer. Glucose uptake was analyzed using flow cytometry on a CytoFLEX Flow Cytometer, and the data were processed using Cytexpert and FlowJo software. Cells were gated on singlets and live cells.

### Lactate detection assay

Lactate levels were measured using the Beyotime Lactate Assay Kit according to the manufacturer's instructions. Briefly, cell culture supernatants or tissue samples were prepared and incubated with the lactate assay reagent. The reaction mixture was incubated at 37 °C for a specified duration, and the absorbance was measured at 450 nm using a microplate reader. The lactate concentration was determined by comparing the absorbance values to a standard curve generated using known lactate concentrations.

### Isolation and purification of exosomes

Exosomes were extracted from the cell culture medium through differential centrifugation. To reduce the risk of exogenous exosome contamination, the experimental cells were cultured for 48 h in a medium supplemented with depleted extracellular vesicle FBS from XP Biomed. The harvested cell culture medium was subjected to successive centrifugation steps: first at 300 g for 10 min at 4°C, then at 2,000 g for 20 min, and finally at 10,000 g for 30 min to remove microvesicles. The supernatant was collected and filtered (0.2 μm). Subsequently, the supernatant was centrifuged at 120,000 g for an additional 70 min, and the pellet was purified via centrifugation in PBS at 120,000 g for 70 min. Precipitated extracellular vesicles were reconstituted in PBS and stored at -80°C for subsequent applications. All centrifugations were performed at 4 °C. The quantification of exosomes was based on the exosome content by using the Micro BCA Protein Assay Kit (Thermo SCIENTIFIC).

### Flow cytometry

Macrophage markers were detected by staining the fixed and permeabilized cells with CD11B-BV510, CD68-PerCP, CD86-APC, and CD206-PE antibodies at 4 °C for 30 min. T-cell markers were detected with FVS510 for live/dead staining, followed by incubation with CD45-APC-Cy7, CD3-APC, CD8-PerCP-Cy5.5, IFN-PE, and PD-1-BV421 antibodies at 4°C for 30 min. After fixation and permeabilization, intracellular staining was performed. The stained cells were then analyzed on the Beckman CytoFLEX flow cytometer so as to determine the proportion of the indicated cell populations.

### Primers and antibodies

All primers and antibodies used in this study are listed in Supplementary Table 1 and Supplementary Table 2.

### Statistical analysis

Statistical analyses were performed using GraphPad Prism 8.0 (GraphPad Software, La Jolla, CA, USA) and SPSS 21.0 (IBM SPSS Statistics for Windows, version 21.0, Armonk, NY, USA). To ensure reproducibility and reliability, a minimum of three biological replicates were included for quantitative analyses, while all animal experiments were conducted with at least six independent biological replicates. The data were presented as the mean ± standard deviation (SD) in bar graphs. The selection of statistical tests was guided by the distribution and characteristics of the data. Shapiro-Wilk test was performed to assess normality. Student's *t*-test was applied for two-group comparisons of normally distributed data, while one-way ANOVA with Tukey's post-hoc test was performed for multiple groups. For non-normally distributed data, the Mann-Whitney U-test and Kruskal-Wallis test were performed for two-group and multi-group comparisons, respectively. Categorical variables were analyzed using the Chi-square test or Fisher's exact test, depending on the expected cell counts. Fisher's exact test was applied when any expected frequency fell below the threshold required for the Chi-square test assumptions. Two-way ANOVA was performed to evaluate the interaction effects between two independent variables on a continuous outcome. Survival analysis was conducted using Kaplan-Meier curves, with comparisons assessed via the log-rank test. Pearson's correlational analysis was performed to assess the relationships between continuous variables. All statistical analyses were performed using two-sided tests, with a significance threshold set at *P* < 0.05 for all comparisons.

Further details on the methodologies used in this study are provided in the Supplementary Materials and methods.

## Results

### CCT2 is essential in HCC tumorigenesis and indicates poor prognosis

To systematically identify TRiC subunits with potential oncogenic relevance in HCC, we first conducted differential expression analyses (tumor vs. adjacent normal tissues; *P* < 0.05, |log2FC| > log2(1.5)) across six independent GEO HCC datasets (GSE25599, GSE124535, GSE101432, GSE105130, GSE112705, and GSE122482) **(Supplementary Fig. 1)**. Intersection of the differentially expressed genes across these datasets yielded a robust set of consistently dysregulated genes, and subsequent overlap with known TRiC subunits identified four candidates, CCT2, CCT3, CCT5, and CCT6A **(Supplementary Fig. 1)**. We next evaluated the expression and clinical relevance of these four candidates using the HCCDB online database (http://lifeome.net/database/hccdb). Among them, only CCT2 and CCT3 displayed consistent and significant upregulation in HCC tissues compared with non-tumor controls across multiple independent cohorts (**Supplementary Table 3 - 6**). We prioritized CCT2 for further investigation because, despite its consistent upregulation and strong clinical relevance in HCC, its mechanistic contribution to HCC progression has not been systematically characterized, warranting in-depth functional and mechanistic analyses.

**Figure 1:**
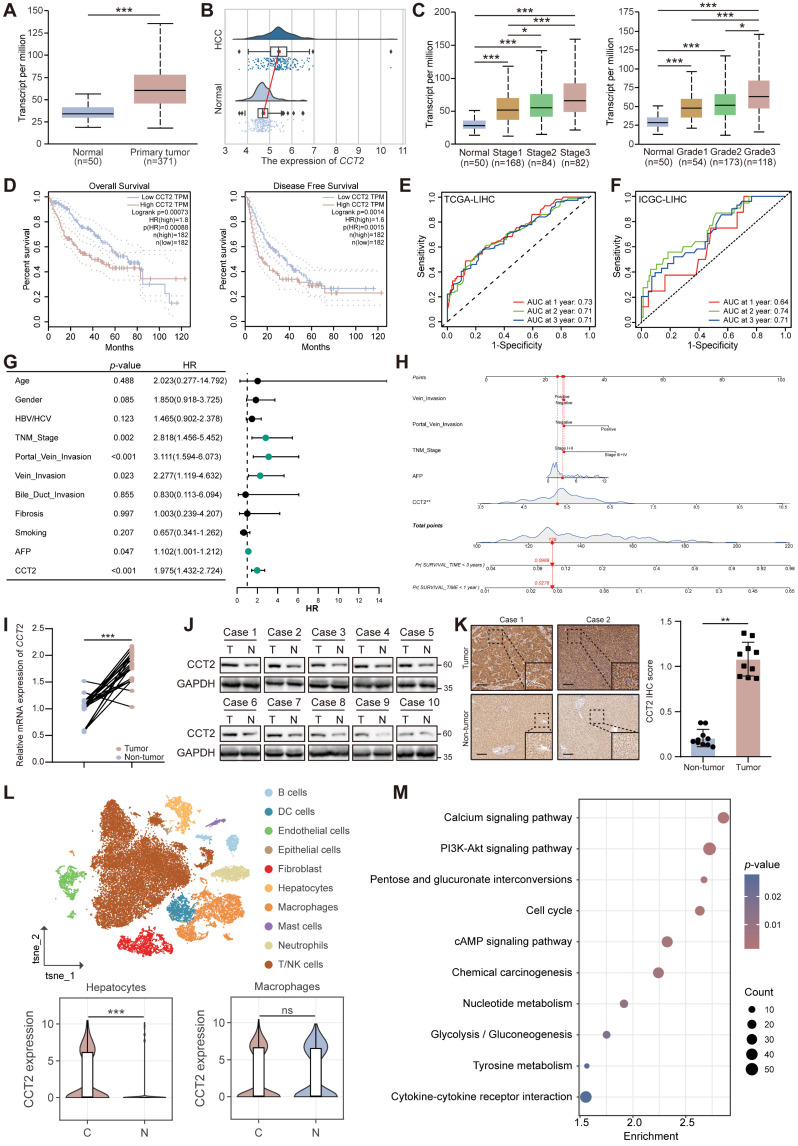
**CCT2 is essential in HCC tumorigenesis and indicates poor prognosis. (A)** CCT2 mRNA expression in HCC tissues (Primary tumor) and normal liver tissues (Normal) of the TCGA-LIHC cohort, data retrieved from the UALCAN database. **(B)** CCT2 mRNA expression in HCC tissues and normal liver tissues of the ICGC-LIHC cohort (*P* < 0.001). **(C)** CCT2 mRNA expression in HCC tissues at different tumor stages and tumor grades of the TCGA-LIHC cohort, data retrieved from the UALCAN database. **(D)** Kaplan-Meier curves for overall survival (OS) and disease-free survival (DFS) based on CCT2 mRNA expression, data retrieved from the GEPIA database. **(E)** ROC curves confirming the prognostic predictive reliability of CCT2 expression in HCC patients in TCGA-LIHC cohort. **(F)** ROC curves confirming the prognostic predictive reliability of CCT2 expression in HCC patients in ICGC-LIHC cohort. **(G)** Univariate Cox regression analyses of prognostic predictive factors of HCC. **(H)** A nomogram showing multivariate Cox regression analyses of the prognostic factors of HCC. **(I)** mRNA expression of CCT2 in tumor and corresponding normal tissues from the Zhongnan cohort analyzed by quantitative polymerase chain reaction (qPCR) (n = 23). **(J)** Protein expression of CCT2 in tumor and corresponding normal tissues from the Zhongnan cohort analyzed by western blot analysis (n = 10). **(K)** Immunohistochemistry for CCT2 in tumor tissues and normal tissues from the Zhongnan cohort (n = 10) (Scale bar, 200 μm). **(L)** Classification of cell clusters and CCT2 mRNA expression in the indicated cell populations in cancerous (C) and normal (N) tissues based on single-cell RNA sequencing data from HCC and adjacent normal tissues (n = 4). **(M)** KEGG analysis of differentially expressed genes from RNA sequencing (RNA-seq) data of oeNC and oeCCT2 Huh7 cells. Differences were analyzed by Student's t-test or ANOVA. After ANOVA, Tukey's post-hoc test was used for multiple comparisons. Data are presented as mean ± SD. *P < 0.05, ** P < 0.01, *** P < 0.001.

To clarify the oncogenic relevance of CCT2 in HCC, we analyzed its expression using the UALCAN portal (http://ualcan.path.uab.edu) and independent datasets from the International Cancer Genome Consortium (ICGC). CCT2 was significantly upregulated in HCC tissues and positively correlated with tumor stage, grade, and invasion depth **(Fig. 1A-C; Supplementary Table 7)**. Kaplan-Meier analysis demonstrated that elevated CCT2 expression was significantly associated with reduced overall and disease-free survival **(Fig. 1D; Supplementary Fig. 2A)**, highlighting its prognostic relevance in HCC.

To further evaluate the prognostic value of CCT2 in HCC, we performed ROC curve analyses, which demonstrated that CCT2 expression had a reliable predictive performance for patient prognosis **(Fig. 1E-F)**. We also assessed the association between CCT2 expression and conventional clinical prognostic parameters using both univariate and multivariate Cox regression analyses **(Fig. 1G-H)**. The results revealed that high CCT2 expression was an independent risk factor for poor overall survival in HCC patients.

To validate these observations, CCT2 mRNA and protein levels were assessed in paired HCC and adjacent non-tumor tissues from Zhongnan Hospital of Wuhan University. The results confirmed significantly elevated CCT2 expression in HCC tissues **(Fig. 1I-K; Supplementary Table 8)**. Single-cell RNA sequencing of four paired HCC and adjacent tissues revealed that CCT2 was predominantly expressed in malignant HCC cells, with no significant difference observed in macrophages, the most abundant immune subset **(Fig. 1L; Supplementary Fig. 2B-D)**. CCT2 expression was also markedly higher in liver cancer cell lines than in normal liver cell lines **(Supplementary Fig. 2E-F)**, reinforcing its oncogenic role in HCC cells.

To investigate the molecular basis of CCT2-driven tumorigenesis, we overexpressed CCT2 in Huh7 cells and conducted transcriptome profiling with Kyoto Encyclopedia of Genes and Genomes (KEGG) analysis, revealing activation of oncogenic pathways such as PI3K-Akt signaling, cell cycle, and glycolysis/gluconeogenesis **(Fig. 1M; Supplementary Fig. 2G)**. KEGG analysis of The Cancer Genome Atlas (TCGA) Liver Hepatocellular Carcinoma (LIHC) data further demonstrated positive correlations between CCT2 expression and multiple tumor-associated pathways **(Supplementary Fig. 2H)**, supporting its role as a driver of HCC progression.

### CCT2 contributes to HCC tumorigenesis and progression

**Figure 2:**
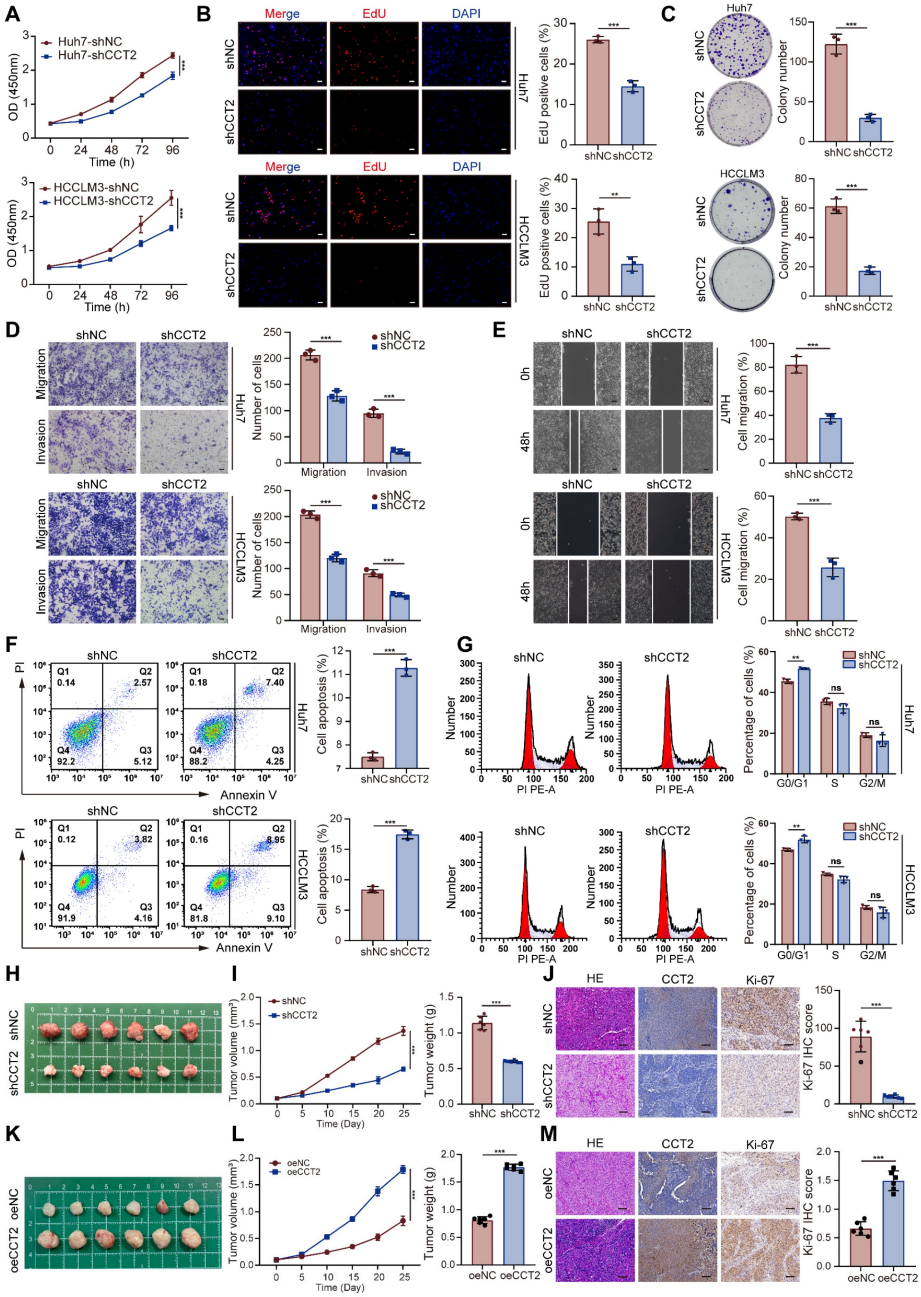
** CCT2 contributes to HCC tumorigenesis and progression. (A)** CCK-8 assay for cell viability assessment of shNC and shCCT2 Huh7 cells and HCCLM3 cells (n = 5). **(B)** EdU assay for cell proliferation assessment of shNC- and shCCT2-transfected Huh7 and HCCLM3 cells (n = 3). **(C)** Clonogenic assay for colony formation ability assessment of shNC- and shCCT2-transfected Huh7 and HCCLM3 cells (n = 3). **(D)** Transwell assay for the assessment of invasion and migration abilities of shNC- and shCCT2-transfected Huh7 and HCCLM3 cells (n = 3; scale bar, 100 μm). **(E)** Wound healing assay for migration assessment of shNC- and shCCT2-transfected Huh7 and HCCLM3 cells (n = 3; scale bar, 100 μm). **(F)** Flow cytometry for apoptosis analysis of shNC- and shCCT2-transfected Huh7 and HCCLM3 cells (n = 3). **(G)** Flow cytometry for cell cycle analysis of shNC- and shCCT2-transfected Huh7 cells (n = 3). **(H)** Subcutaneous xenografts in BALB/c nude mice implanted with shNC- and shCCT2-transfected HCCLM3 cells (n = 6). **(I)** Tumor volume and tumor weight of subcutaneous xenografts in BALB/c nude mice implanted with shNC- and shCCT2-transfected HCCLM3 cells (n = 6). **(J)** Immunohistochemistry for HE staining, CCT2, and Ki-67 in the xenografts (n = 6; scale bar, 200 μm). **(K)** Subcutaneous xenografts in BALB/c nude mice implanted with oeNC- and oeCCT2-transfected Hep3B cells (n = 6). **(L)** Tumor volume and tumor weight of subcutaneous xenografts in BALB/c nude mice implanted with oeNC- and oeCCT2-transfected Hep3B cells (n = 6). **(M)** Immunohistochemistry for HE staining, CCT2, and Ki-67 in the CCT2-overexpressed xenografts (n = 6; scale bar, 200 μm). Differences were analyzed by Student's t-test or ANOVA. After ANOVA, Tukey's post-hoc test was used for multiple comparisons. Three biological replicates were performed for cell experiments. Data are presented as mean ± SD. ns, not significant, *P < 0.05, ** P < 0.01, *** P < 0.001.

To investigate the functional role of CCT2 in HCC, we performed *in vitro* and *in vivo* experiments. Stable knockdown of CCT2 in Huh7 and HCCLM3 cells markedly impaired cell viability and proliferative capacity, as evidenced by reduced CCK-8 activity, EdU incorporation, and colony formation **(Fig. 2A-C)**. Attempts to generate CCT2-knockout HCC cells using CRISPR/Cas9 were unsuccessful, as complete CCT2 deletion resulted in cell death. Conversely, CCT2 overexpression in Hep3B cells enhanced proliferation compared to controls **(Supplementary Fig. 3A-C)**.

Transwell and wound healing assays demonstrated that CCT2 depletion impaired, whereas overexpression enhanced, migratory and invasive capacities **(Fig. 2D-E; Supplementary Fig. 3D-E)**. Transcriptomic analysis implicated CCT2 in cell cycle regulation and oncogenic signaling. Flow cytometry revealed that CCT2 depletion induced apoptosis and G0/G1 arrest **(Fig. 2F-G)**, whereas overexpression reduced apoptosis and increased the G2-phase population **(Supplementary Fig. 3F-G)**, indicating its role in promoting cell cycle progression.

To validate these findings *in vivo*, we conducted subcutaneous xenograft assays using CCT2-knockdown HCCLM3 cells in BALB/c nude mice. CCT2 silencing markedly suppressed tumor growth, as reflected by reduced tumor volume and weight **(Fig. 2H-I)**. Pathological analysis revealed decreased Ki-67 expression and less aggressive histology in shCCT2 tumors **(Fig. 2J)**. In contrast, CCT2 overexpression enhanced tumor growth, Ki-67 expression, and histological malignancy **(Fig. 2K-M)**, reinforcing its oncogenic role in HCC. Collectively, these results confirmed CCT2 as a key mediator of HCC tumorigenesis and underscored its potential as a therapeutic target.

### CCT2 promotes glycolysis in HCC cells via interaction with ALDOA

**Figure 3:**
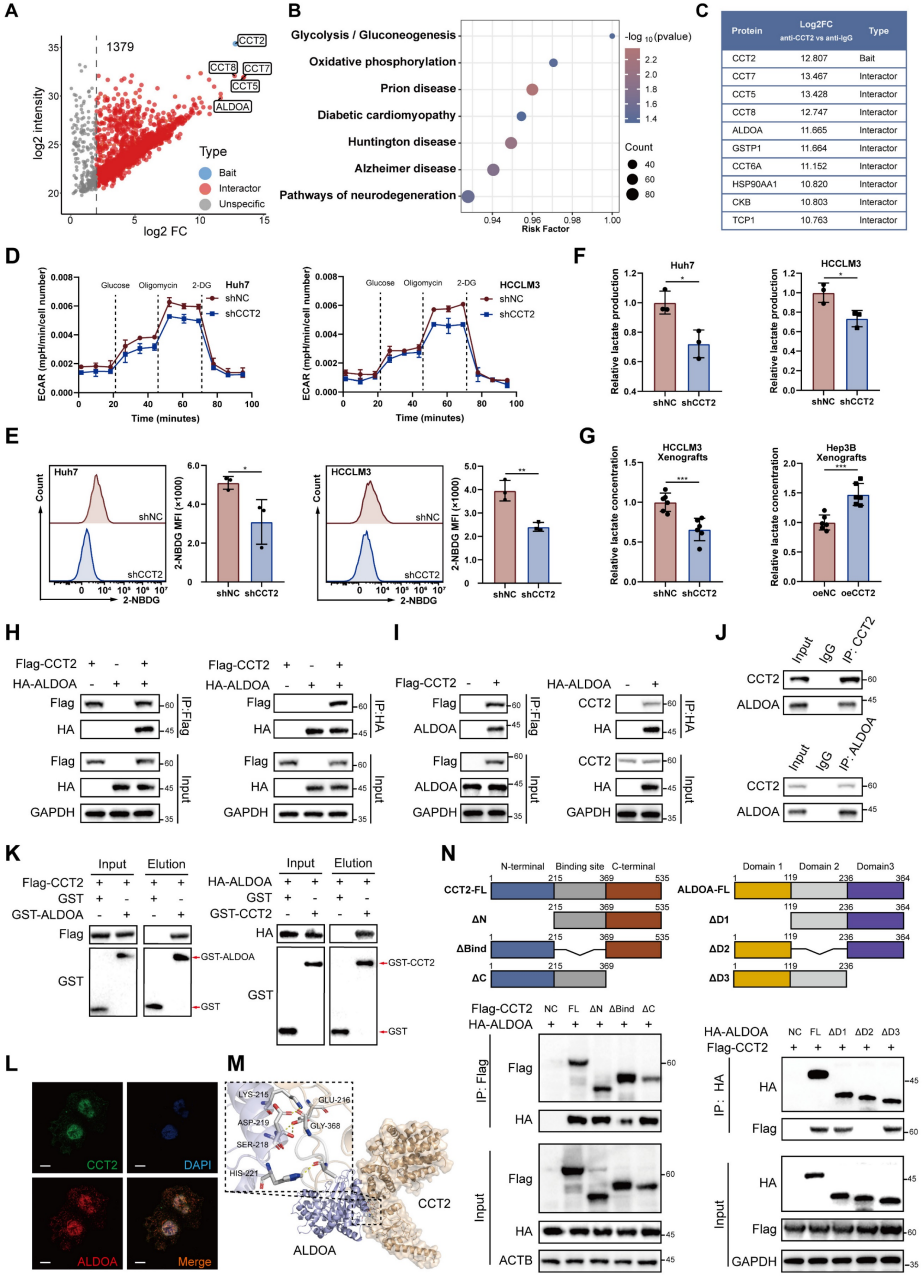
** CCT2 promotes glycolysis in HCC cells via interaction with ALDOA. (A)** Volcano plot of the interactors of CCT2 detected by co-IP/MS (1379 interactors). **(B)** KEGG pathway analysis of CCT2 interactors identified by co-IP/MS. **(C)** Top-10 interactors of CCT2 based on Log2FC (anti-CCT2 vs. anti-IgG). **(D)** Extracellular acidification rate (ECAR) assay for glycolytic metabolism assessment of shNC- and shCCT2-transfected Huh7 and HCCLM3 cells (n = 3). **(E)** Glucose uptake assay of shNC- and shCCT2-transfected Huh7 cells and HCCLM3 cells (n = 3). **(F)** Lactate detection assay in the medium of shNC- and shCCT2-transfected Huh7 cells and HCCLM3 cells (n = 3). **(G)** Lactate levels in subcutaneous xenografts in BALB/c nude mice implanted with shNC- and shCCT2-transfected HCCLM3 cells (n = 6) and in subcutaneous xenografts in BALB/c nude mice implanted with oeNC- and oeCCT2-transfected Hep3B cells (n = 6). **(H)** Exogenous co-IP followed by western blot to detect the interaction between CCT2 (Flag-CCT2) and ALDOA (HA-ALDOA) in HEK293T cells. **(I)** Semi-exogenous co-IP followed by western blot to detect the interaction between CCT2 (Flag-CCT2/CCT2) and ALDOA (HA-ALDOA/ALDOA) in Huh7 cells. **(J)** Endogenous co-IP followed by western blot to detect the interaction between CCT2 and ALDOA in Huh7 cells. **(K)** GST pulldown assays for detecting direct interactions between CCT2 and ALDOA proteins. **(L)** Immunofluorescence staining of CCT2 and ALDOA in Huh7 cells (Scale bar, 5 μm). **(M)** Molecular docking analysis of CCT2 and ALDOA using HDOCK (confidence score: 0.9118). **(N)** Schematic diagram of human CCT2 domains and strategy for generating CCT2 deletion mutants and co-IP experiments in HEK293T cells to assess interactions between ALDOA and CCT2 fragments. Differences were analyzed by Student's t-test or ANOVA. After ANOVA, Tukey's post-hoc test was used for multiple comparisons. Three biological replicates were performed for cell experiments. Data are presented as mean ± SD. *P < 0.05, ** P < 0.01, *** P < 0.001.

CCT2 functions as a chaperone protein regulating protein folding and stability through interactions with client proteins 26. To investigate its oncogenic mechanism in HCC, we performed co-immunoprecipitation followed by mass spectrometry (co-IP/MS), identifying 1,379 potential interactors **(Fig. 3A-C; Supplementary Fig. 4A)**. KEGG enrichment analysis ranked glycolysis/gluconeogenesis as the top pathway, suggesting CCT2 involvement in glycolytic regulation **(Fig. 3B)**. To assess its functional impact, we measured ECAR in Huh7 and HCCLM3 cells, showing that CCT2 knockdown significantly impaired glycolytic activity, along with reduced glucose uptake and lactate production **(Fig. 3D-F)**. *In vivo*, CCT2 silencing decreased tumor lactate levels, whereas overexpression increased them, further supporting its role in promoting glycolysis **(Fig. 3G)**.

Among the glycolysis-related proteins identified in our co-IP/MS analysis, aldolase A (ALDOA), a key glycolytic enzyme 29, emerged as the most abundant interactor **(Fig. 3C)**. We hypothesized that CCT2 regulates glycolysis via interaction with ALDOA. Co-IP assays in HEK293T cells co-transfected with Flag-CCT2 and HA-ALDOA confirmed their physical interaction, which was further validated by semi-exogenous and endogenous co-IP in Huh7 cells **(Fig. 3H-J)**. GST pulldown assays demonstrated direct binding between CCT2 and ALDOA **(Fig. 3K)**. Immunofluorescence staining showed strong co-localization of CCT2 and ALDOA **(Fig. 3L)**, indicating a functional interaction.

To elucidate the structural basis of the CCT2-ALDOA interaction, computational docking predicted a high-confidence binding (score = 0.9118) **(Fig. 3M)**. Domain-mapping co-IP assays with Flag-tagged CCT2 and HA-tagged ALDOA mutants showed that deletion of CCT2's substrate-binding domain (ΔBind) markedly reduced binding, while removal of ALDOA's second domain (ΔD2) abrogated the interaction **(Fig. 3N)**. These results demonstrate that specific structural domains mediate the CCT2-ALDOA interaction, which may underlie CCT2-associated glycolytic regulation in HCC cells.

### CCT2 enhances HCC glycolysis and tumorigenesis via ALDOA stabilization

**Figure 4:**
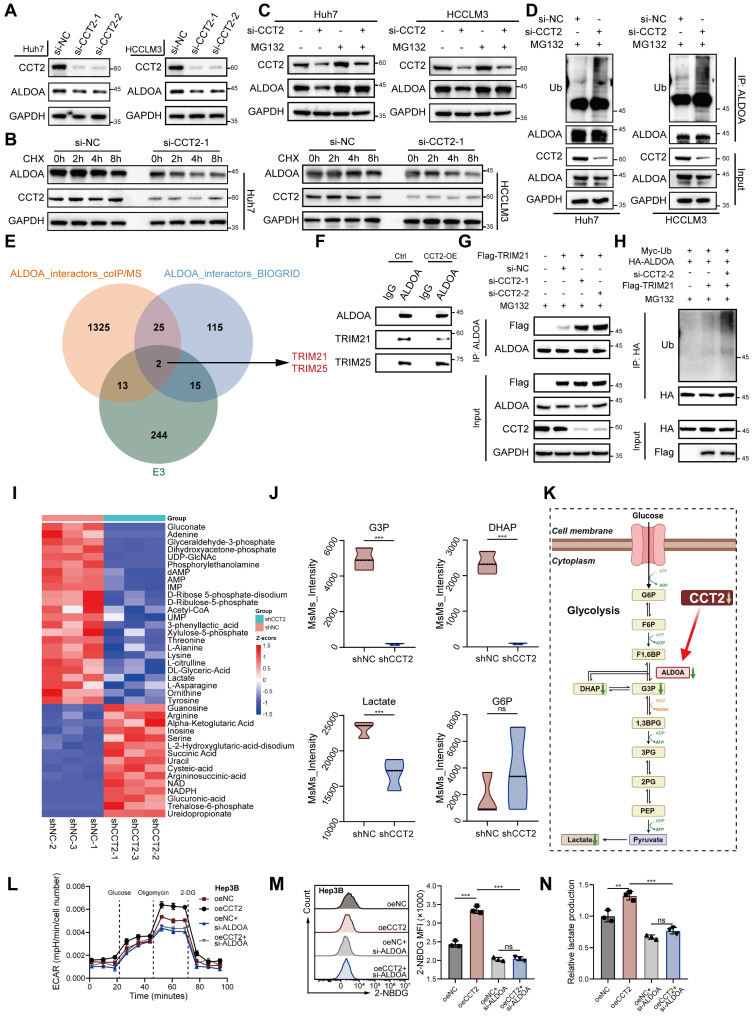
** CCT2 enhances HCC glycolysis and tumorigenesis via ALDOA stabilization. (A)** Protein expression of CCT2 and ALDOA in Huh7 and HCCLM3 cells transfected with si-NC, si-CCT2-1, and si-CCT2-2, determined by western blot analysis. **(B)** Cycloheximide (CHX) (200 μg/mL) chase assay to assess ALDOA degradation, determined by western blot analysis in Huh7 and HCCLM3 cells transfected with si-NC and si-CCT2-1. **(C)** Protein expression of CCT2 and ALDOA in Huh7 and HCCLM3 cells transfected with si-NC and si-CCT2-1, treated with or without MG132 (15 μM), determined by western blot analysis. **(D)** Ubiquitination of ALDOA detected by western blot analysis of ubiquitin, CCT2, and ALDOA in Huh7 and HCCLM3 cells transfected with si-NC or si-CCT2. **(E)** Venn diagram showing overlap between ALDOA-interacting proteins identified by co-immunoprecipitation coupled with mass spectrometry and known ALDOA interactors from the BioGRID database, identifying TRIM21 and TRIM25 as candidate E3 ubiquitin ligases. **(F)** Co-IP assays showing interactions between ALDOA and TRIM21 or TRIM25 in control and CCT2-overexpressing cells. **(G)** Co-IP assays showing increased binding of TRIM21 to ALDOA upon CCT2 knockdown in Huh7 cells. **(H)** Ubiquitination assay of ALDOA in cells transfected with indicated constructs, showing that CCT2 knockdown enhances TRIM21-mediated ubiquitination of ALDOA. **(I)** Targeted metabolite analysis in shNC- and shCCT2-transfected Huh7 cells (n = 3) (Metabolites with *P* < 0.05 are shown). **(J)** Quantification of G3P, DHAP, lactate, and G6P detected by targeted metabolite analysis in shNC- and shCCT2-transfected Huh7 cells (n = 3). **(K)** Diagram illustrating significant changes in the indicated metabolites in the glycolysis pathway, identified after CCT2 knockdown by targeted metabolite analysis, with ALDOA shown (green downward arrows indicate significantly downregulated metabolites or proteins). **(L)** ECAR assay for glycolytic metabolism assessment of oeNC- and oeCCT2-transfected Hep3B cells with or without si-ALDOA treatment (n = 3). **(M)** Glucose uptake assay in oeNC- and oeCCT2-transfected Hep3B cells with or without si-ALDOA treatment (n = 3). **(N)** Lactate detection assay in oeNC- and oeCCT2-transfected Hep3B cells with or without si-ALDOA treatment (n = 3). Differences were analyzed by Student's t-test or ANOVA. After ANOVA, Tukey's post-hoc test was used for multiple comparisons. Three biological replicates were performed for cell experiments. Data are presented as mean ± SD. CHX, cycloheximide; Ub, ubiquitin; G6P, glucose-6-phosphate; F6P, fructose-6-phosphate; F1,6BP, fructose-1,6-bisphosphate; G3P, glyceraldehyde-3-phosphate; DHAP, dihydroxyacetone phosphate; 1,3BPG, 1,3-bisphosphoglycerate; 3PG, 3-phosphoglycerate; 2PG, 2-phosphoglycerate; PEP, phosphoenolpyruvate. ns, not significant, *P < 0.05, ** *P* < 0.01, *** *P* < 0.001.

Following confirmation of the CCT2-ALDOA interaction, we investigated whether CCT2 regulates ALDOA. CCT2 modulated ALDOA protein levels without affecting its mRNA expression **(Fig. 4A; Supplementary Fig. 5A-B)**. In contrast, knockdown of CCT4 or CCT5—adjacent TRiC subunits—had no effect on ALDOA levels, and ALDOA knockdown did not affect CCT2 expression **(Supplementary Fig. 5C-E)**, suggesting a specific post-transcriptional regulatory effect of CCT2 on ALDOA.

Given CCT2's function as a molecular chaperone, we examined whether it regulates ALDOA stability. Cycloheximide chase assays showed that CCT2 knockdown reduced ALDOA half-life while overexpression prolonged it, indicating that CCT2 stabilizes ALDOA by protecting it from degradation **(Fig. 4B; Supplementary Fig. 5F)**. MG132 treatment restored ALDOA levels in CCT2-depleted cells, whereas chloroquine had no effect, and CCT2 knockdown significantly increased ALDOA ubiquitination **(Fig. 4C-D; Supplementary Fig. 5G)**, confirming that CCT2 prevents its proteasomal degradation.

To further elucidate the mechanism by which CCT2 regulates ALDOA ubiquitination, we sought to identify the E3 ubiquitin ligase responsible for ALDOA degradation and to investigate whether CCT2 modulates this process. Co-IP/MS of ALDOA-associated proteins, combined with interrogation of published ALDOA interactors in the BioGRID database (https://thebiogrid.org, identified two candidate E3 ligases, TRIM21 and TRIM25 **(Fig. 4E)**. Subsequent co-immunoprecipitation assays confirmed that both TRIM21 and TRIM25 physically interact with ALDOA **(Fig. 4F)**. However, overexpression of CCT2 markedly reduced the interaction between ALDOA and TRIM21, whereas the association between ALDOA and TRIM25 remained unchanged **(Fig. 4F)**, suggesting that CCT2 may influence ALDOA ubiquitination by selectively affecting its interaction with TRIM21.

Consistently, CCT2 knockdown significantly enhanced the binding of TRIM21 to ALDOA, further indicating that CCT2 impairs the ALDOA-TRIM21 interaction **(Fig. 4G)**. Functional ubiquitination assays showed that TRIM21 promotes ubiquitination of ALDOA, and depletion of CCT2 further increased ALDOA ubiquitination, supporting that CCT2 regulates ALDOA ubiquitination in a TRIM21-dependent manner **(Fig. 4H)**. Further ubiquitin linkage analysis revealed that TRIM21 predominantly induces K48-linked polyubiquitination of ALDOA **(Supplementary Fig. 5I)**, and site-specific ubiquitination assays demonstrated that TRIM21 facilitates ubiquitination at K13, K87, and K139 residues of ALDOA **(Supplementary Fig. 5J)**. Together, these results demonstrate that CCT2 stabilizes ALDOA by disrupting its interaction with the E3 ligase TRIM21, thereby suppressing TRIM21-mediated ubiquitination.

Metabolomic profiling further revealed that CCT2 depletion reduced key glycolytic intermediates, including glyceraldehyde-3-phosphate (G3P), dihydroxyacetone phosphate (DHAP), and lactate, with a slight but non-significant increase in glucose-6-phosphate (G6P) **(Fig. 4I-K; Supplementary Fig. 5H)**, consistent with ALDOA's metabolic role 29. To further confirm that CCT2 exerts its glycolytic and oncogenic effects through ALDOA, ALDOA knockdown reversed the metabolic changes induced by CCT2 overexpression, including ECAR, glucose uptake, and lactate production **(Fig. 4L-N)**. It also reduced CCT2-driven cell proliferation and migration **(Supplementary Fig. 5K-M)**. Collectively, these findings demonstrate that CCT2 promotes glycolysis and tumorigenesis in HCC by stabilizing ALDOA, highlighting the CCT2-ALDOA axis as a potential therapeutic target.

### CCT2 promotes M2-like TAM infiltration in the TME and suppresses the efficacy of PD-1 mAb blockade

**Figure 5:**
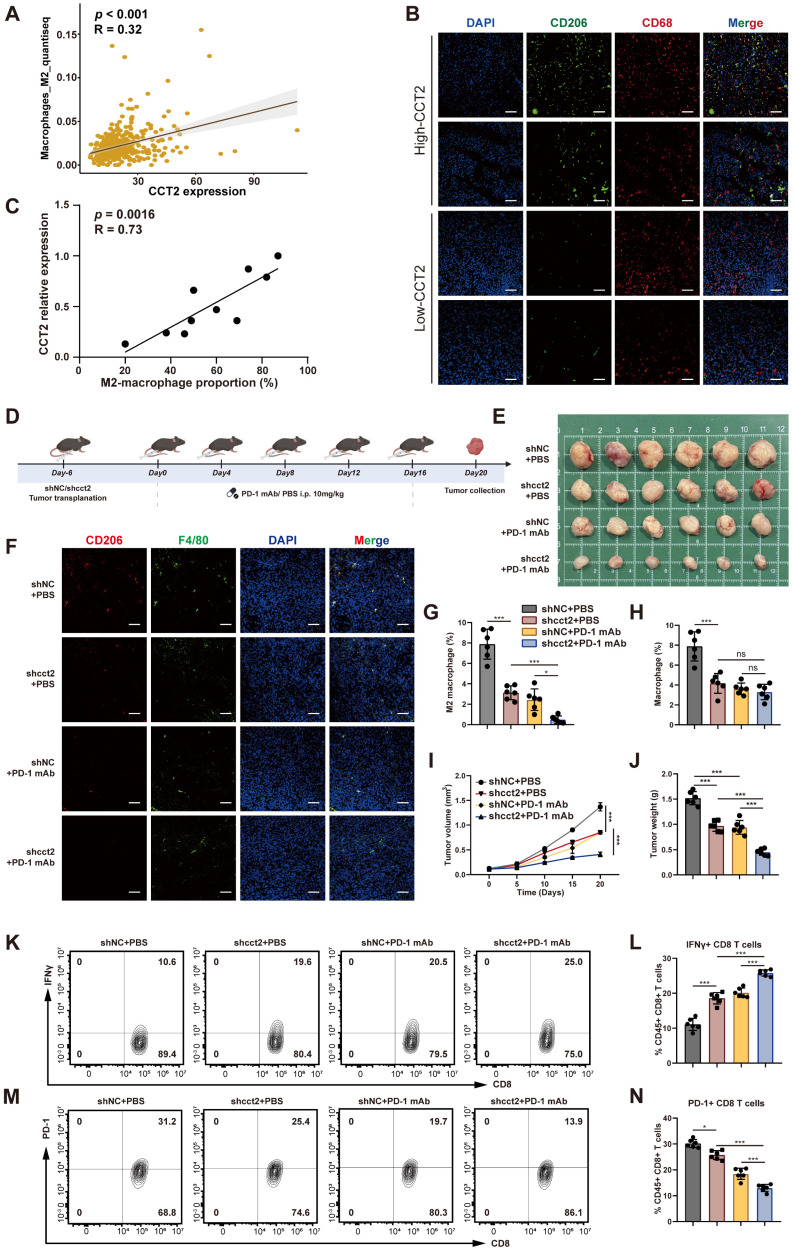
** CCT2 promotes M2-like TAM infiltration in the TME and suppresses the efficacy of PD-1 mAb blockade. (A)** Pearson's correlation analysis between CCT2 expression and M2-like macrophage infiltration based on TCGA-LIHC data. **(B)** Representative images of immunofluorescence staining for CD206 (green) and CD68 (red) in tumor tissues from HCC patient groups classified as Low-CCT2 and High-CCT2 based on the median CCT2 expression level (Scale bar, 100 μm). **(C)** Pearson's correlation analysis between CCT2 expression and M2-like macrophage (CD206^+^ CD68^+^) infiltration in tumor tissues from HCC patients. **(D)** Schematic diagram of subcutaneous xenograft implantation using shNC- and shCCT2-transfected Hep53.4 cells in C57BL/6 mice, with or without PD-1 mAb (10 mg/kg). **(E)** Subcutaneous xenografts in C57BL/6 mice implanted with shNC- or shCCT2-transfected Hep53.4 cells, with or without PD-1 mAb treatment (n = 6). **(F)** Representative images of immunofluorescence staining for CD206 (red) and F4/80 (green) in subcutaneous xenograft tissues (Scale bar, 100 μm). **(G)** Quantification of M2 macrophages (CD206⁺ F4/80⁺) in subcutaneous xenograft tissues (n = 6). **(H)** Quantification of total macrophages (F4/80⁺) in subcutaneous xenograft tissues (n = 6). **(I)** Tumor volume of subcutaneous xenografts in the indicated groups (n = 6). **(J)** Tumor weight of subcutaneous xenografts in the indicated groups (n = 6). **(K, L)** Flow cytometry analysis of IFNγ⁺ CD8⁺ T cells extracted from subcutaneous xenografts in the indicated groups (n = 6). **(M, N)** Flow cytometry analysis of PD-1⁺ CD8⁺ T cells extracted from subcutaneous xenografts in the indicated groups (n = 6). Group labels in (G), (H), (J), (L), and (N) are consistent. Differences were analyzed by Student's t-test or ANOVA. After ANOVA, Tukey's post-hoc test was used for multiple comparisons. Three biological replicates were performed for cell experiments. Data are presented as mean ± SD. ns, not significant, *P < 0.05, ** P < 0.01, *** P < 0.001.

Given that CCT2 stabilizes ALDOA to promote glycolysis and lactate production in HCC cells—and considering lactate's role in immune modulation and the reported link between CCT2 and tumor immunity—we investigate whether CCT2 affect HCC TME 23,30. QuanTIseq deconvolution of TCGA-LIHC data revealed a significant positive correlation between CCT2 expression and M2 macrophage infiltration—an immunosuppressive subtype—further confirmed by immunofluorescence in human HCC tissues **(Fig. 5A-C; Supplementary Fig. 6A)**.

Given the established association between M2 macrophage polarization and reduced responsiveness to PD-1 blockade 31, we analyzed the GEO dataset GSE215011 comprising HCC patients treated with anti-PD-1 therapy and observed that CCT2 expression was significantly higher in non-responders than in responders **(Supplementary Fig. 6B)**. We next examined the impact of CCT2 on the tumor immune microenvironment and the therapeutic efficacy of PD-1 mAb in murine xenograft models **(Fig. 5D-E)**. Silencing CCT2 reduced M2 macrophage infiltration, and its combination with PD-1 mAb further decreased M2 levels **(Fig. 5F-H)**. Tumor growth was markedly suppressed in the CCT2-knockdown group, with enhanced response when combined with PD-1 blockade **(Fig. 5I-J)**. Flow cytometry revealed increased IFNγ+CD8+T cells and reduced PD-1+ CD8+ T cells **(Fig. 5K-N)**, indicating improved cytotoxic activity and reduced exhaustion. Consistently, in an orthotopic HCC mouse model, CCT2 knockdown significantly reduced M2 macrophage infiltration **(Supplementary Fig. 6C)**, decreased the proportion of PD-1⁺ CD8⁺ T cells **(Supplementary Fig. 6D)**, suppressed tumor burden, and prolonged overall survival **(Supplementary Fig. 6E-G)**. Notably, under PD-1 mAb treatment, tumors with CCT2 knockdown exhibited significantly reduced tumor burden compared with control tumors, demonstrating that CCT2 depletion enhances responsiveness to PD-1 blockade. Collectively, these findings identify CCT2 as an important modulator of the immunosuppressive TME in HCC and a contributor to reduced therapeutic efficacy of PD-1 blockade.

### CCT2 promotes M2 polarization via lactate production from HCC cells

**Figure 6:**
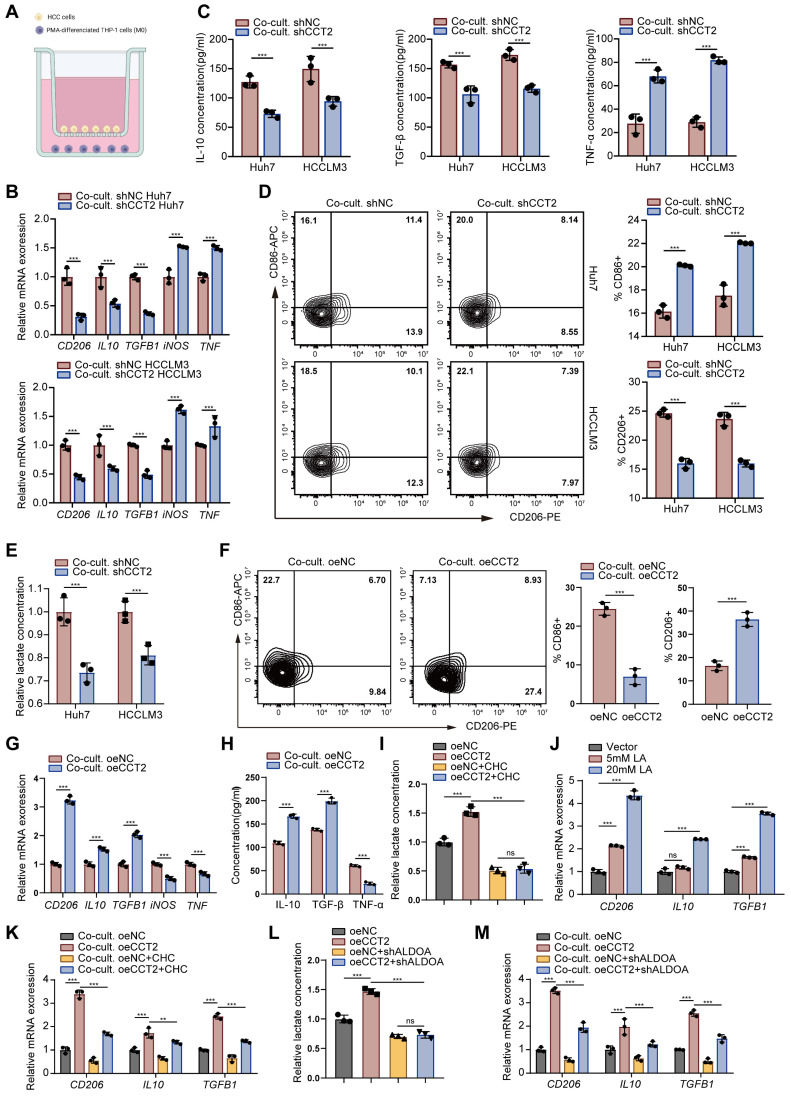
** CCT2 promotes M2 polarization via lactate production from HCC cells. (A)** Schematic diagram of the co-culture system. **(B)** mRNA expression levels of *CD206*, *IL10*, *TGFB1*, *iNOS*, and *TNF* in PMA-treated THP1 cells co-cultured with the indicated HCC cells, determined by qPCR (n = 3). **(C)** ELISA assays for IL-10, TGF-β, and TNF-α in the indicated culture supernatants (n = 3). **(D)** Flow cytometry analysis of the proportion of CD86⁺ (M1) and CD206⁺ (M2) cells in the indicated THP1 cell groups (n = 3). **(E)** Lactate detection in the indicated cell culture supernatants (n = 3). **(F)** Flow cytometry analysis of the proportion of M1 and M2 cells in the indicated THP1 cell groups (n = 3). **(G)** mRNA expression levels of *CD206*, *IL10*, *TGFB1*, *iNOS*, and *TNF* in PMA-treated THP1 cells co-cultured with the indicated HCC cells, determined by qPCR (n = 3). **(H)** ELISA assays for IL-10, TGF-β, and TNF-α in the indicated culture supernatants (n = 3). **(I)** Lactate detection in the indicated cell culture supernatants (n = 3). **(J)** mRNA expression levels of *CD206*,* IL10*, and *TGFB1* in THP1 cells treated with lactate (LA) at different concentrations, determined by qPCR (n = 3). **(K)** mRNA expression levels of *CD206*, *IL10*, and *TGFB1* in PMA-treated THP1 cells co-cultured with oeNC- and oeCCT2-transfected Huh7 cells, with or without CHC, determined by qPCR (n = 3). **(L)** Lactate detection in the indicated cell culture supernatants (n = 3). **(M)** mRNA expression levels of *CD206*,* IL10*, and *TGFB1* in PMA-treated THP1 cells co-cultured with oeNC- and oeCCT2-transfected Huh7 cells, with or without shALDOA, determined by qPCR (n = 3). Differences were analyzed by Student's t-test or ANOVA. After ANOVA, Tukey's post-hoc test was used for multiple comparisons. Three biological replicates were performed for cell experiments. Data are presented as mean ± SD. Co-cult., co-culture with; CHC, chlorohydroxycinnamate. ns, not significant, *P < 0.05, ** P < 0.01, *** P < 0.001.

To investigate whether CCT2-induced changes in HCC cells directly promote M2 polarization, a co-culture system was utilized **(Fig. 6A)**. Co-culturing PMA-stimulated M0 THP1 cells (referred to as macrophages) with CCT2-knockdown HCC cells suppressed M2 markers (*CD206*, *IL10*, *TGFB1*) and increased M1 markers (*iNOS*, *TNF*) **(Fig. 6B)**. ELISA confirmed reduced IL-10 and TGF-β and elevated TNF-α in the conditioned medium **(Fig. 6C)**. Flow cytometry revealed fewer CD206+ and more CD86+ macrophages, alongside decreased lactate levels **(Fig. 6D-E)**. Conversely, CCT2 overexpression enhanced M2 polarization and lactate accumulation **(Fig. 6F-I)**, suggesting a link between lactate and macrophage polarization.

To evaluate this, macrophages exposed to increasing lactate concentrations showed enhanced M2 polarization **(Fig. 6J)**. Inhibiting lactate secretion in HCC cells with chlorohydroxycinnamate (CHC), a lactate secretion inhibitor 32, attenuated CCT2-induced M2 polarization and lactate levels, though not completely **(Fig. 6K)**. Moreover, ALDOA knockdown in HCC cells partially reversed CCT2-driven polarization **(Fig. 6L-M)**. Collectively, these results indicate that CCT2 promotes M2 macrophage polarization in HCC partially via lactate production.

### Both exosomes and lactate contribute to CCT2-mediated M2 polarization

**Figure 7:**
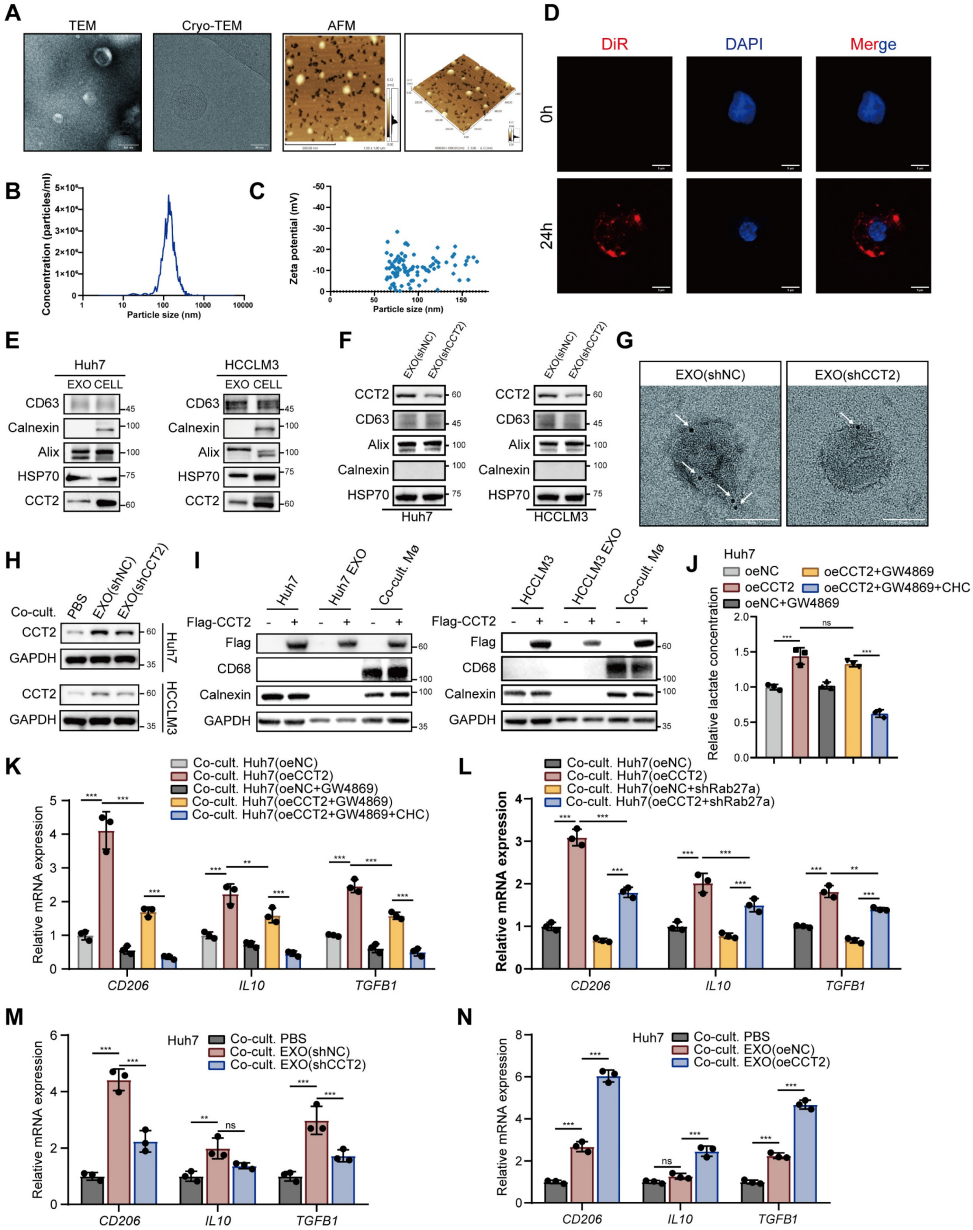
** Both exosomes and lactate contribute to CCT2-mediated M2 polarization. (A)** Transmission electron microscopy (TEM) imaging (Scale bar, 200 nm), cryo-electron microscopy (cryo-EM) (Scale bar, 50 nm) and atomic force microscopy (AFM) imaging (Scale bar, 500 nm) of the morphology of extracted exosomes. **(B)** Nanoparticle tracking analysis (NTA) showing the size distribution and concentration of exosomes. **(C)** Zeta potential measurements of the extracted exosomes. **(D)** Exosome THP1-uptake evaluation at 0 h and 24 h using DiR staining (Scale bar, 5 μm). **(E)** Western blot analysis detecting CCT2, exosomal markers (CD63, HSP70, and Alix), and the endoplasmic reticulum marker Calnexin in exosomes and cell lysates from Huh7 and HCCLM3 cells. **(F)** Western blot analysis detecting CCT2 in exosomes from indicated Huh7 and HCCLM3 cells. **(G)** Immunogold labeling of exosomes derived from shNC- and shCCT2-transfected Huh7 cells, using an anti-CCT2 antibody followed by secondary antibodies conjugated to 4 nm gold particles (indicated by white arrows) (Scale bar, 50 nm). **(H)** Protein expression of CCT2 in PMA-treated THP1 cells directly co-cultured with PBS or exosomes derived from Huh7 and HCCLM3 cells, determined by western blot analysis. **(I)** Western blot analysis detecting Flag-CCT2, macrophage marker CD68, and Calnexin in Huh7 and HCCLM3 cells, their corresponding exosomes (Huh7/HCCLM3 EXO), and PMA-treated THP1 cells co-cultured with the corresponding exosomes (Co-cult. Mø). To dissect the relative contribution of exosome-mediated signaling and lactate-dependent effects, tumor cells were pretreated with the exosome secretion inhibitor GW4869, alone or in combination with the lactate transport inhibitor CHC, prior to co-culture with PMA-treated THP1 cells. **(J)** Lactate levels in culture supernatants of oeNC- and oeCCT2-transfected Huh7 cells under the indicated conditions, including GW4869 pretreatment and combined GW4869+CHC treatment in oeCCT2 cells (n = 3). **(K)** mRNA expression levels of *CD206*, *IL10*, and *TGFB1* in PMA-treated THP1 cells co-cultured with oeNC- or oeCCT2-transfected Huh7 cells under the indicated conditions, including GW4869 pretreatment and combined GW4869+CHC treatment in oeCCT2 cells, determined by qPCR (n = 3). **(L)** mRNA expression levels of *CD206*,* IL10*, and *TGFB1* in PMA-treated THP1 cells co-cultured with oeNC- or oeCCT2-transfected Huh7 cells, with or without Rab27a knockdown in tumor cells, determined by qPCR (n = 3). **(M-N)** mRNA expression levels of *CD206*, *IL10*, and *TGFB1* in PMA-treated THP1 cells directly co-cultured with PBS or exosomes derived from indicated cells, determined by qPCR (n = 3). Differences were analyzed by Student's t-test or ANOVA. After ANOVA, Tukey's post-hoc test was used for multiple comparisons. Three biological replicates were performed for cell experiments. Data are presented as mean ± SD. Co-cult., co-culture with; CHC, chlorohydroxycinnamate. ns, not significant, *P < 0.05, ** P < 0.01, *** P < 0.001.

CCT2 contributes to M2 polarization partly through lactate production, suggesting additional mechanisms. Given that CCT2 is present in exosomes and that exosomes modulate macrophage polarization within the TME 33, we hypothesized that CCT2 might also influence macrophage polarization via exosomes. To investigate this, we isolated exosomes from HCC cells and confirmed their morphology by transmission electron microscopy (TEM), cryo-electron microscopy (Cryo-TEM), and atomic force microscopy (AFM) **(Fig. 7A)**. Nanoparticle tracking analysis (NTA) and Zeta potential analysis validated their size and stability **(Fig. 7B-C)**. Uptake assays showed efficient internalization by macrophages **(Fig. 7D)**. Western blotting and immuno-electron microscopy (IEM) confirmed CCT2 in exosomes, which was reduced upon CCT2 knockdown **(Fig. 7E-G)**. Co-culture with exosomes derived from CCT2-knockdown HCC cells resulted in reduced CCT2 levels in recipient macrophages, while overexpression of Flag-CCT2 in donor cells led to the detection of Flag-CCT2 in macrophages, confirming that CCT2 is transferred from tumor cells to macrophages via exosomal delivery **(Fig. 7H-I)**. Inhibition of exosome release with GW4869, an exosome secretion inhibitor 34, attenuated CCT2-induced M2 polarization, co-inhibition with CHC further suppressed it **(Fig. 7J-K)**. Consistently, genetic inhibition of exosome release by Rab27a knockdown in HCC cells also reduced the effect of CCT2 on inducing M2 polarization in macrophages **(Fig. 7L; Supplementary Fig. 7A)**. To further determine whether exosomes alone are sufficient to modulate macrophage polarization, purified exosomes were directly co-cultured with macrophages. Macrophages exhibited lower M2 polarization when co-cultured with exosomes from CCT2-knockdown cells and higher polarization with those from CCT2-overexpressing cells, when equal amounts of exosomes were used **(Fig. 7M-N; Supplementary Fig. 7B)**. Notably, when exosomes were isolated from HCC cells in which lactate export was inhibited by CHC, their CCT2-mediated M2-polarizing effect on macrophages remained unchanged, indicating that lactate does not affect the intrinsic polarization activity of exosomes **(Supplementary Fig. 7C)**.

To evaluate the relative contribution of lactate signaling and exosomal communication to CCT2-induced macrophage polarization, we inhibited lactate export using CHC and blocked exosome release using GW4869 in the co-culture system with oeCCT2 HCC cells, either individually or in combination. Inhibition of either pathway alone partially reduced the expression of M2 markers, including *CD206*, *IL10*, and *TGFB1*, whereas combined inhibition resulted in a more pronounced suppression than either single treatment **(Supplementary Fig. 7D)**. These findings indicate that lactate-mediated and exosome-mediated signaling contribute through separable but complementary mechanisms to CCT2-driven macrophage polarization.

### CCT2-mediated GSTP1 transfer via exosomes promotes M2 polarization through AKT-NF-κB signaling axis

**Figure 8:**
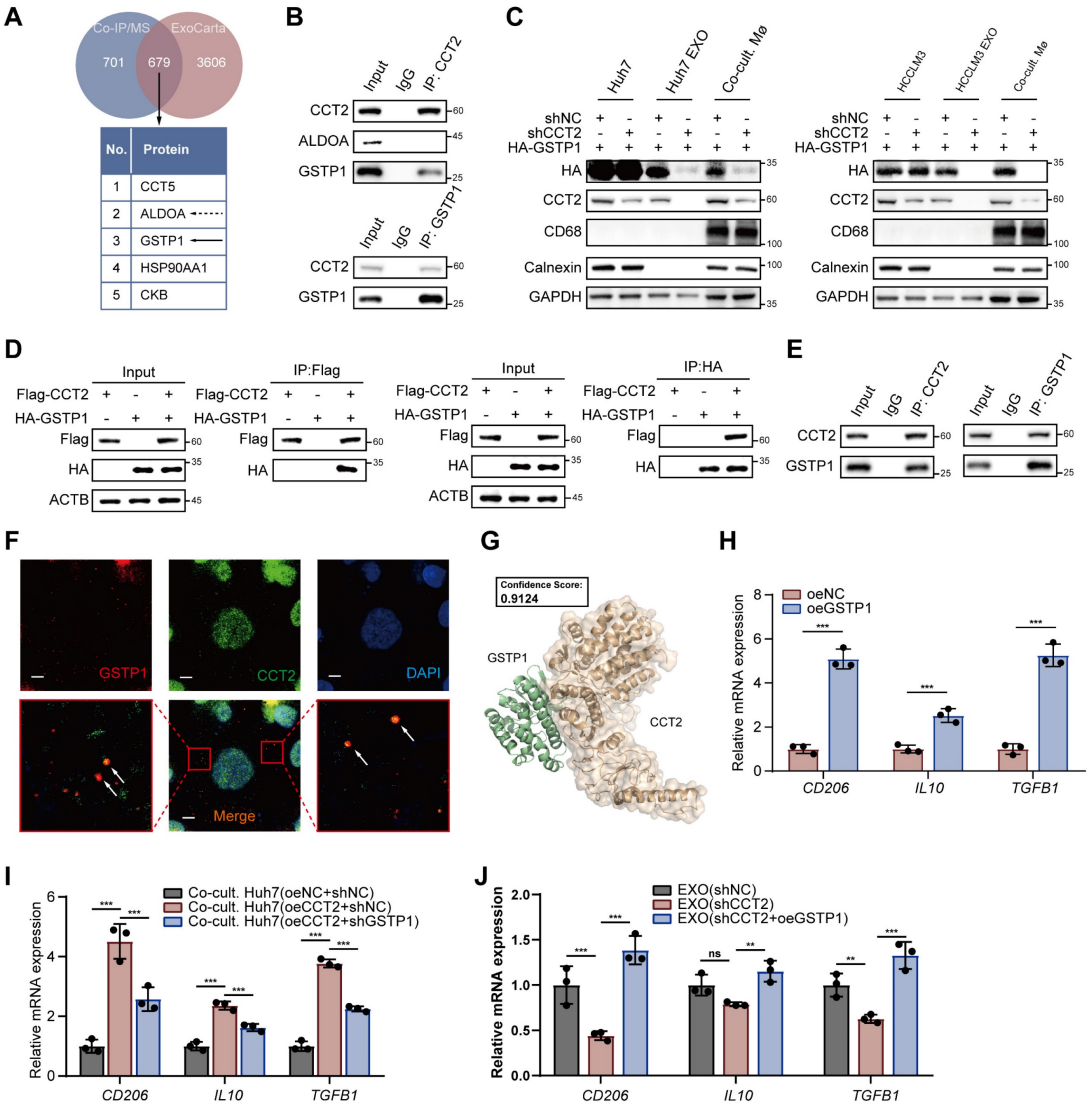
** CCT2-mediated GSTP1 Transfer via Exosomes Promotes M2 Polarization. (A)** Diagram illustrating the intersection of CCT2 interactors identified by co-immunoprecipitation coupled with mass spectrometry and exosomal proteins identified in humans from ExoCarta. **(B)** Endogenous co-IP followed by western blot analysis to detect the interaction between CCT2 and ALDOA or GSTP1 in exosomes derived from Huh7 cells. **(C)** Western blot analysis detecting HA-GSTP1, macrophage marker CD68, and Calnexin in Huh7 and HCCLM3 cells, their corresponding exosomes (Huh7/HCCLM3 EXO), and PMA-treated THP1 cells co-cultured with the corresponding exosomes (Co-cult. Mø). **(D)** Exogenous co-IP followed by western blot analysis to detect the interaction between CCT2 (Flag-CCT2) and GSTP1 (HA-GSTP1) in HEK293T cells. **(E)** Endogenous co-IP followed by western blot to detect the interaction between CCT2 and GSTP1 in Huh7 cells. **(F)** Immunofluorescence staining of CCT2 and GSTP1 in Huh7 cells (Scale bar, 5 μm). **(G)** Molecular docking analysis of CCT2 and GSTP1 using HDOCK. **(H)** mRNA expression levels of *CD206*, *IL10*, and *TGFB1* in PMA-treated THP1 cells transduced with oeNC and oeGSTP1 vectors, determined by qPCR (n = 3). **(I)** mRNA expression levels of *CD206*, *IL10*, and *TGFB1* in PMA-treated THP1 cells co-cultured with the indicated Huh7 cells, determined by qPCR (n = 3). **(J)** mRNA expression levels of *CD206*, *IL10*, and *TGFB1* in PMA-treated THP1 cells treated with exosomes derived from shNC, shCCT2, or shCCT2 cells with GSTP1 overexpression (shCCT2 + oeGSTP1), determined by qPCR (n = 3). Differences were analyzed by Student's t-test or ANOVA. After ANOVA, Tukey's post-hoc test was used for multiple comparisons. Three biological replicates were performed for cell experiments. Data are presented as mean ± SD. Co-cult., co-culture with; EXO, exosomes. ns, not significant, *P < 0.05, ** P < 0.01, *** P < 0.001.

Our findings indicate that CCT2 promotes M2 polarization through exosome-mediated intercellular communication from HCC cells. However, direct overexpression of CCT2 in macrophages did not significantly affect M2 polarization **(Supplementary Fig. 8A)**, nor did it induce detectable changes in macrophage glycolytic activity **(Supplementary Fig. 8B-C)**, suggesting an indirect mechanism involving exosomal cargo. By intersecting proteins identified in CCT2 co-IP/MS with entries from the ExoCarta exosome database **(Fig. 8A)**, ALDOA emerged as the top interactor but was scarcely present in exosomes and poorly internalized by macrophages **(Fig. 8B; Supplementary Fig. 6D)**, excluding it as a mediator.

Glutathione S-transferase P (GSTP1), the second most abundant interactor, exhibited a significant interaction with CCT2 in exosomes **(Fig. 8B)**. Notably, knockdown of CCT2 in HCC cells did not significantly alter GSTP1 protein or mRNA levels in donor cells **(Fig. 8C; Supplementary Fig. 8E-F)**, but markedly reduced GSTP1 enrichment in exosomes and decreased GSTP1 levels in recipient macrophages after exosome co-culture **(Fig. 8C)**. Conversely, CCT2 overexpression did not affect intracellular GSTP1 expression in HCC cells but significantly increased GSTP1 loading into exosomes and its subsequent accumulation in macrophages, indicating that CCT2 facilitates GSTP1 transfer to macrophages via exosomes **(Supplementary Fig. 8E)**. Moreover, CCT2 and GSTP1 co-localized in vesicle-like structures in Huh7 cells, and their interaction was further supported by co-immunoprecipitation and docking analysis **(Fig. 8D-G)**. Functionally, GSTP1 overexpression in macrophages enhanced M2 marker expression, while GSTP1 knockdown in HCC cells impaired CCT2-induced M2 polarization **(Fig. 8H-I; Supplementary Fig. 6G-H).** Importantly, modulation of GSTP1 in HCC cells did not affect CCT2-induced lactate production, indicating that GSTP1 regulates macrophage polarization independently of tumor cell glycolysis **(Supplementary Fig. 6G)**. Furthermore, GSTP1 overexpression rescued the impaired M2-polarizing activity of exosomes from CCT2-knockdown cells in exosome-macrophage co-culture assays **(Fig. 8J; Supplementary Fig. 8I)**, indicating that CCT2 promotes M2 polarization primarily through GSTP1 transfer.

**Figure 9:**
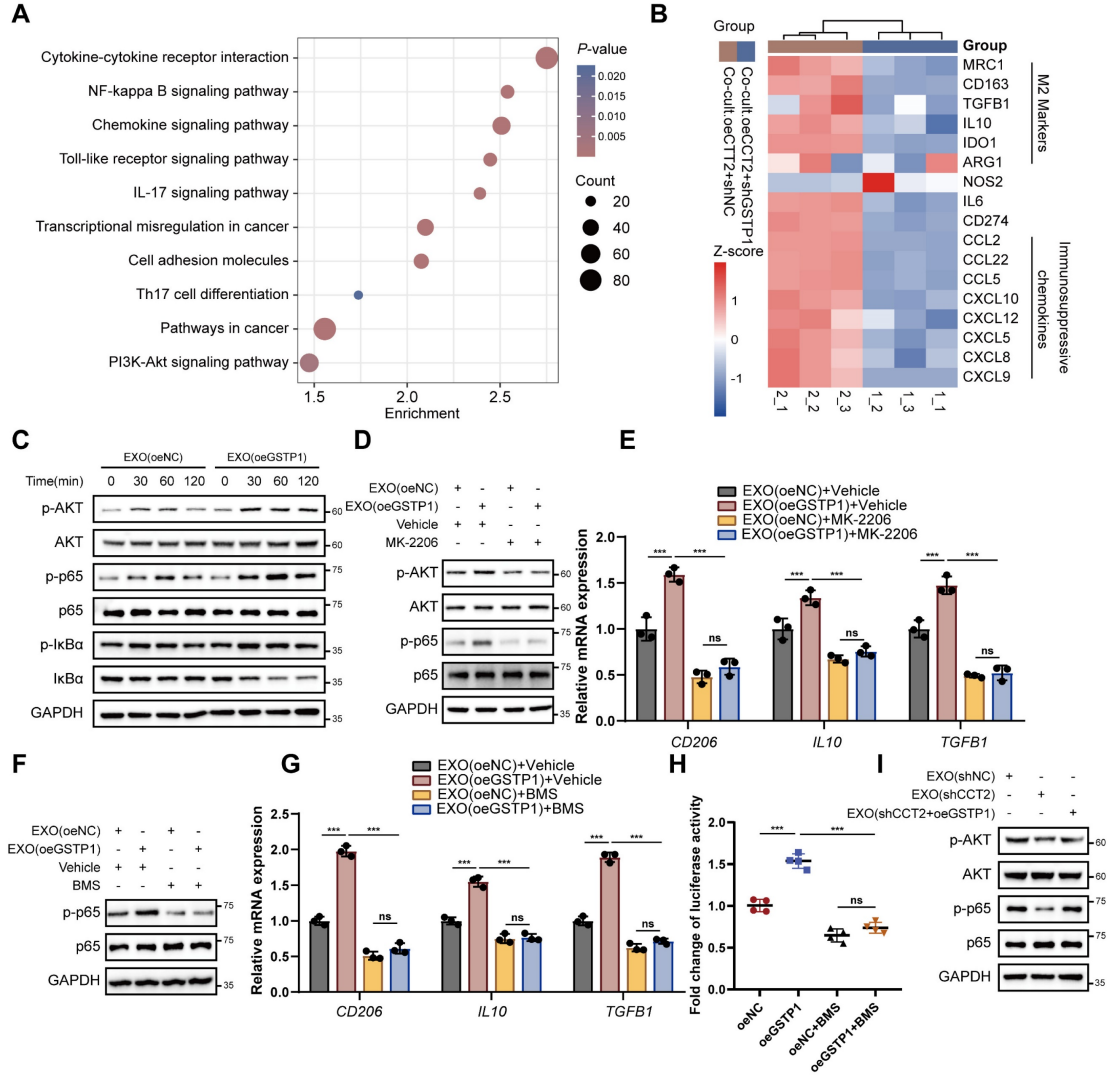
** Exosomal GSTP1 activates the AKT-NF-κB signaling axis to promote M2 polarization. (A)** KEGG pathway analysis of differentially expressed genes from RNA-seq data of PMA-treated THP1 cells co-cultured with the indicated Huh7 cells. **(B)** Heatmap of the expression levels of the indicated genes from RNA-seq data of PMA-treated THP1 cells co-cultured with the indicated Huh7 cells. **(C)** Time-course western blot analysis of p-AKT, p-p65, and p-IκBα in PMA-treated THP1 cells treated with exosomes derived from oeNC or oeGSTP1 HCC cells. **(D)** Western blot analysis of AKT and NF-κB signaling in THP1 cells treated with exosomes in the presence or absence of the AKT inhibitor MK-2206 (5 μM). **(E)** qPCR analysis of *CD206*, *IL10*, and *TGFB1* in THP1 cells treated as in (D) (n = 3). **(F)** Western blot analysis of p65 signaling in THP1 cells treated with exosomes in the presence or absence of the IKK inhibitor BMS-345541 (1 μM).** (G)** qPCR analysis of *CD206*, *IL10*, and *TGFB1* in THP1 cells treated as in (F) (n = 3). **(H)** NF-κB luciferase reporter activity in THP1 cells overexpressing GSTP1, with or without BMS-345541 treatment. Differences were analyzed by Student's t-test or ANOVA. After ANOVA, Tukey's post-hoc test was used for multiple comparisons. Three biological replicates were performed for cell experiments. Data are presented as mean ± SD. Co-cult., co-culture with; EXO, exosomes. ns, not significant, *P < 0.05, ** P < 0.01, *** P < 0.001.

Transcriptomic profiling of macrophages co-cultured with CCT2-overexpressing HCC cells revealed that GSTP1 knockdown in HCC cells altered polarization-related pathways, including NF-κB and PI3K-Akt, and reduced M2 markers and immunosuppressive genes, some of which were also elevated in GSTP1-overexpressing macrophages **(Fig. 9A-B; Supplementary Fig. 9A)**. Western blot analysis further confirmed pathway activation, showing that exosomal GSTP1 increased phosphorylation of AKT, p65, and IκBα, whereas STAT3 phosphorylation remained unchanged **(Fig. 9C; Supplementary Fig. 9B)**. Pharmacological inhibition of AKT with MK2206 abolished activation of both AKT and NF-κB signaling induced by exosomes derived from GSTP1-overexpressing HCC cells and suppressed exosome-mediated M2 polarization **(Fig. 9D-E)**. In addition, inhibition of IκB signaling with the IκB kinase (IKK) inhibitor BMS-345541 in macrophages abrogated the pro-polarization effect of exosomal GSTP1 **(Fig. 9F-G)**. Consistently, overexpression of GSTP1 in macrophages activated an NF-κB reporter **(Fig. 9H)**, confirming that GSTP1 promotes M2 polarization through activation of the AKT-NF-κB signaling axis. Finally, in exosome-macrophage co-culture assays, restoration of GSTP1 expression rescued the impaired activation of AKT-NF-κB signaling caused by CCT2 knockdown **(Fig. 9I)**, further supporting that GSTP1 modulates CCT2-mediated exosomal activation of the AKT-NF-κB signaling axis. Together, these results demonstrate that CCT2 promotes M2 polarization through exosome-mediated transfer of GSTP1 to macrophages, leading to activation of the AKT-NF-κB signaling pathway.

## Discussion

HCC is one of the most prevalent and lethal malignancies, posing a major threat to human health 1,2. The limited understanding of the complex molecular mechanisms underlying tumor-TME crosstalk during hepatocarcinogenesis hinders the effectiveness of current therapies, leading to poor patient outcomes. Identifying key drivers of HCC tumorigenesis is therefore critical for developing targeted therapeutic strategies.

CCT2, a subunit of the TRiC complex, has recently emerged as a crucial regulator of tumorigenesis 25,26,33, yet its role in HCC remains poorly defined. Elevated CCT2 expression in HCC tissues has been associated with tumor stage and patient survival 35, but the underlying molecular mechanisms require further elucidation. While these findings suggest an oncogenic role for CCT2 in HCC, mechanistic insights remain limited compared to other cancers. In colorectal cancer, CCT2 has been implicated in hypoxia-driven oncogenic signaling, correlating with the Hedgehog pathway effector Gli-1, a key determinant of patient prognosis 25. This suggests a potential link between CCT2 and adaptive tumor responses to hypoxia, a hallmark of the TME. Similarly, in glioblastoma, CCT2 has been reported to promote tumor progression by interacting with KRAS proteins, thereby modulating oncogenic signaling 26. Despite these insights, the specific molecular mechanisms through which CCT2 contributes to HCC progression remain largely unexplored.

In this study, analysis of HCC patient data from TCGA and ICGC databases confirmed a significant correlation between CCT2 expression, HCC tumorigenesis, and patient prognosis, consistent with previous findings. Immunohistochemical analysis further validated the increased expression of CCT2 in HCC tissues. Single-cell RNA sequencing analysis revealed that CCT2 is predominantly expressed in HCC cells. Pathway enrichment analysis demonstrated that CCT2 is associated with multiple carcinogenesis-related pathways, reinforcing its role in HCC progression. Functional experiments* in vitro* and *in vivo* further confirmed that CCT2 promotes malignant phenotypes in HCC cells, highlighting its potential as a therapeutic target.

Given the molecular functions of CCT2 36, co-IP/MS analysis revealed a strong association between CCT2 and glycolytic pathways in HCC cells, as further supported by ECAR, glucose uptake, and lactate production assays. To identify the key mediator of CCT2-driven glycolysis, we analyzed MS data and found that ALDOA was the most abundant glycolysis-related interactor of CCT2. ALDOA, a key glycolytic enzyme, has been reported to promote glycolysis and proliferation in HCC cells 29. Consistently, our findings demonstrated that CCT2 directly interacts with and stabilizes ALDOA. Truncation-based co-IP assays further revealed that CCT2's substrate-binding site specifically interacts with ALDOA's domain 2, the region responsible for its enzymatic activity 37. Since ALDOA catalyzes the reversible conversion of fructose-1,6-bisphosphate into DHAP and G3P, which are essential for glycolysis 38, metabolomic profiling showed that CCT2 knockdown significantly reduced DHAP, G3P, and lactate levels in HCC cells, consistent with ALDOA's metabolic role. Functional assays further demonstrated that ALDOA knockdown reversed CCT2-induced glycolysis enhancement and oncogenic effects, supporting a central role of ALDOA in mediating CCT2-driven metabolic reprogramming in HCC.

While no prior studies have directly linked CCT2 to cellular metabolism, research on other CCT subunits suggests their roles in metabolic regulation. Knockdown of CCT subunits via siRNA has been shown to alter lipid composition, reprogram metabolism toward lipid dependence, and increase peroxisomal and mitochondrial activity 39. In HCC, CCT3 promotes tumor growth mainly by modulating lipid metabolism 40. From the perspective of glycolysis, CCT3 has also been implicated in lung adenocarcinoma, where its depletion suppresses tumor growth and metastasis by reducing ATP production through glycolysis 41. Additionally, CCT4 knockdown enhances the sensitivity of esophageal squamous cell carcinoma cells to cisplatin by modulating glucose metabolism and regulating key glycolytic enzymes such as hexokinase-2, lactate dehydrogenase A (LDHA), and phosphoglycerate mutase 1 42. These studies indicate that individual CCT subunits can influence glycolytic activity mainly at the pathway or multi-enzyme regulation level. In contrast, our study reveals a more direct and specific mechanism whereby CCT2 stabilizes the key glycolytic enzyme ALDOA at the protein level, thereby sustaining glycolytic flux in HCC cells.

Our study revealed that CCT2 promotes lactate accumulation in the extracellular environment, consistent with its role in glycolysis. As a byproduct of glycolysis and a weak acid, lactate plays a critical role in the TME by modulating immune responses 23. It influences several immune cell types essential for anti-tumor immunity 43,44. Additionally, CCT2 has been correlated with tumor immunity 30. Based on these findings, we investigated whether CCT2 affects anti-tumor immunity in HCC. Immune infiltration analysis and tissue detection in HCC samples revealed a significant correlation between CCT2 expression and M2 macrophage infiltration. TAMs are mainly classified into M1 and M2 subtypes based on function 45. M1 macrophages, activated by LPS, GM-CSF, or IFN-γ, exhibit a pro-inflammatory phenotype and exert antitumoral effects. In contrast, M2 macrophages, particularly M2a, are induced by IL-4 and IL-13, promoting immunosuppression and tumor progression. Their distinct roles in the tumor microenvironment make them key targets for cancer therapy. CCT2 knockdown led to reduced M2 macrophage infiltration and increased CD8^+^ T cell activity. Co-culture experiments further confirmed that HCC cell-intrinsic CCT2 promotes M2 macrophage polarization, partially mediated by lactate, aligning with the pro-glycolytic function of CCT2 in HCC cells. Previous studies have shown that tumor-derived lactic acid induces vascular endothelial growth factor expression and promotes M2-like polarization of tumor-associated macrophages 46. Beyond macrophages, lactate modulates other immune cells in the TME, suppressing anti-tumor immunity through multiple mechanisms. Elevated lactate levels drive extracellular acidification, inhibiting cytotoxic T lymphocyte and natural killer cell functions by impairing proliferation, reducing IFN-γ and granzyme B production, and suppressing cytotoxic activity 47,48. Additionally, lactate activates myeloid-derived suppressor cells via HIF-1α and NF-κB signaling, leading to increased secretion of immunosuppressive cytokines such as IL-10 and TGF-β 49. However, the potential role of CCT2 in regulating these immunosuppressive mechanisms remains to be explored further.

Our findings suggest that lactate only partially accounts for CCT2-mediated M2 polarization. The presence of CCT2 in exosomes and the significance of exosomes in the TME provide insights into further investigating their role in CCT2-mediated M2 polarization. Exosomal CCT2 was detected in HCC cell-derived exosomes following successful exosome extraction. GW4869 treatment, which inhibits exosome release, confirmed the role of exosomes in CCT2-driven M2 polarization. Exosome uptake analysis and direct co-culture experiments further demonstrated that exosomal CCT2 contributes to M2 polarization. Recent studies have identified CCT2 as an exosomal protein that suppresses the pro-inflammatory functions of CD4^+^ T cells via JAK2/STAT3 activation in breast cancer cells 33. Notably, exosomal CCT6A derived from cancer-associated fibroblasts has been shown to promote tumorigenesis and chemoresistance in gastric cancer through activation of β-catenin signaling in recipient tumor cells 50. However, in our study, CCT2 knockdown did not significantly alter the JAK2/STAT3 pathway in HCC cells (data not shown), suggesting potential cell-type-specific differences or malignancy-dependent mechanisms. In comparison, our findings indicate that exosomal CCT2 primarily targets macrophages and contributes to immune suppression in HCC, highlighting a CCT2-mediated immunomodulatory mechanism via exosomes.

We further investigated the role of exosomal CCT2 in M2 polarization. Our findings suggest that neither CCT2 nor ALDOA directly mediates this process. Instead, GSTP1, which interacts with CCT2 in exosomes, is transported into macrophages, where it promotes M2 polarization and immunosuppressive functions. Previous studies have shown that GSTP1 inhibits M1 polarization 51 and enhances IL-6 expression in M2-like macrophages 52. Consistently, we found that GSTP1 promotes M2 polarization and increases the expression of IL6 and several immunosuppressive chemokines, reinforcing its immunosuppressive role. Mechanistically, we found that CCT2-mediated GSTP1 transfer to macrophages via exosomes promotes M2 polarization through the AKT-NF-κB signaling axis. The identification of an exosome-dependent GSTP1 pathway, in parallel with CCT2-driven metabolic reprogramming, indicates that CCT2 promotes macrophage M2 polarization through dual mechanisms, providing a rationale for therapeutic strategies targeting both tumor metabolism and exosomal communication in HCC.

Targeting CCT2 as a therapeutic strategy in cancer remains largely unexplored, particularly in HCC. The TRiC complex inhibitor CT20 has been reported to exhibit anti-tumor activity in breast cancer 53. In this context, selectively targeting CCT2 may offer a more precise approach to suppress HCC progression and enhance anti-tumor immunity, given its defined substrate-binding interface and essential role in protein folding. Small-molecule inhibitors targeting the interaction interface between CCT2 and its client proteins could be explored to attenuate CCT2-mediated stabilization of oncogenic substrates such as ALDOA 54. Alternatively, targeted protein degradation strategies, including proteolysis-targeting chimeras (PROTACs), may provide a feasible route to selectively deplete CCT2^55^, thereby simultaneously suppressing tumor-intrinsic glycolysis and exosome-mediated immunosuppressive signaling. In addition to direct CCT2 targeting, pharmacological inhibition of glycolytic pathways or exosome biogenesis may also represent indirect strategies to counteract CCT2-driven macrophage polarization and tumor immune evasion 56,57. At present, selective pharmacological agents targeting CCT2 have not yet been developed or validated in HCC models. Therefore, the translational strategies discussed here are intended to provide a mechanistic and conceptual framework to guide future drug development efforts. Importantly, because CCT2 depletion enhances responsiveness to PD-1 blockade *in vivo*, targeting CCT2-associated pathways may serve as an effective adjuvant strategy to overcome resistance to immune checkpoint inhibitors in HCC.

## Conclusions

This study identifies CCT2 as a key regulator of HCC progression, orchestrating both metabolic reprogramming and immune modulation. We demonstrate that elevated CCT2 expression correlates with poor prognosis in patients with HCC, while its depletion suppresses tumor growth by disrupting glycolytic metabolism. Mechanistically, CCT2 stabilizes ALDOA by impairing TRIM21-mediated ubiquitination, thereby enhancing glycolytic flux and lactate production, which not only promotes tumor proliferation and migration but also drives M2 macrophage polarization. In addition, CCT2-mediated GSTP1 transfer to macrophages via exosomes promotes M2 polarization through activation of the AKT-NF-κB signaling axis. Our findings bridge a critical gap in understanding how tumor cells exploit a common molecular framework to drive both intrinsic malignancy and immune microenvironment remodeling. These insights position CCT2 as a promising therapeutic target, offering new opportunities for disrupting HCC progression and enhancing immunotherapy efficacy.

## Supplementary Material

Supplementary figures and tables.

## Data Availability

The data that support the findings of this study are available from the corresponding author upon reasonable request.

## References

[1] Siegel RL, Giaquinto AN, Jemal A (2024). Cancer statistics, 2024. CA Cancer J Clin.

[2] Siegel RL, Miller KD, Fuchs HE, Jemal A (2021). Cancer Statistics, 2021. CA Cancer J Clin.

[3] Toh MR, Wong EYT, Wong SH (2023). Global Epidemiology and Genetics of Hepatocellular Carcinoma. Gastroenterology.

[4] Rumgay H, Arnold M, Ferlay J (2022). Global burden of primary liver cancer in 2020 and predictions to 2040. J Hepatol.

[5] Huang DQ, Mathurin P, Cortez-Pinto H, Loomba R (2023). Global epidemiology of alcohol-associated cirrhosis and HCC: trends, projections and risk factors. Nat Rev Gastroenterol Hepatol.

[6] Sung H, Ferlay J, Siegel RL (2021). Global Cancer Statistics 2020: GLOBOCAN Estimates of Incidence and Mortality Worldwide for 36 Cancers in 185 Countries. CA Cancer J Clin.

[7] Lu Y, Yang A, Quan C (2022). A single-cell atlas of the multicellular ecosystem of primary and metastatic hepatocellular carcinoma. Nat Commun.

[8] Chen C, Wang Z, Ding Y, Qin Y (2023). Tumor microenvironment-mediated immune evasion in hepatocellular carcinoma. Front Immunol.

[9] Liu Y, Xun Z, Ma K (2023). Identification of a tumour immune barrier in the HCC microenvironment that determines the efficacy of immunotherapy. J Hepatol.

[10] de Visser KE, Joyce JA (2023). The evolving tumor microenvironment: From cancer initiation to metastatic outgrowth. Cancer Cell.

[11] Wang L, Shen K, Gao Z (2024). Melanoma Derived Exosomes Amplify Radiotherapy Induced Abscopal Effect via IRF7/I-IFN Axis in Macrophages. Adv Sci.

[12] He G, Peng X, Wei S (2022). Exosomes in the hypoxic TME: from release, uptake and biofunctions to clinical applications. Mol Cancer.

[13] Hanahan D (2022). Hallmarks of Cancer: New Dimensions. Cancer Discov.

[14] Lian X, Yang K, Li R (2022). Immunometabolic rewiring in tumorigenesis and anti-tumor immunotherapy. Mol Cancer.

[15] Yang F, Hilakivi-Clarke L, Shaha A (2023). Metabolic reprogramming and its clinical implication for liver cancer. Hepatology.

[16] Aoki T, Nishida N, Kurebayashi Y (2025). Molecular Classification of Hepatocellular Carcinoma based on Zoned Metabolic Feature and Oncogenic Signaling Pathway. Clin Mol Hepatol.

[17] Wang S, Zhou L, Ji N (2023). Targeting ACYP1-mediated glycolysis reverses lenvatinib resistance and restricts hepatocellular carcinoma progression. Drug Resist Updat.

[18] Fendt S-M (2024). 100 years of the Warburg effect: A cancer metabolism endeavor. Cell.

[19] Du D, Liu C, Qin M (2022). Metabolic dysregulation and emerging therapeutical targets for hepatocellular carcinoma. Acta Pharm Sin B.

[20] Xu S, Deng K-Q, Lu C (2024). Interleukin-6 classic and trans-signaling utilize glucose metabolism reprogramming to achieve anti- or pro-inflammatory effects. Metabolism.

[21] Tang B, Zhu J, Wang Y (2023). Targeted xCT-mediated Ferroptosis and Protumoral Polarization of Macrophages Is Effective against HCC and Enhances the Efficacy of the Anti-PD-1/L1 Response. Adv Sci.

[22] Yang J, Wang H, Li B (2025). Inhibition of ACSS2 triggers glycolysis inhibition and nuclear translocation to activate SIRT1/ATG5/ATG2B deacetylation axis, promoting autophagy and reducing malignancy and chemoresistance in ovarian cancer. Metabolism.

[23] Zhang A, Xu Y, Xu H (2021). Lactate-induced M2 polarization of tumor-associated macrophages promotes the invasion of pituitary adenoma by secreting CCL17. Theranostics.

[24] Gestaut D, Zhao Y, Park J (2022). Structural visualization of the tubulin folding pathway directed by human chaperonin TRiC/CCT. Cell.

[25] Park SH, Jeong S, Kim BR (2020). Activating CCT2 triggers Gli-1 activation during hypoxic condition in colorectal cancer. Oncogene.

[26] Zhao F, Yao Z, Li Y (2024). Targeting the molecular chaperone CCT2 inhibits GBM progression by influencing KRAS stability. Cancer Lett.

[27] Yu H, Pan J, Zheng S (2023). Hepatocellular Carcinoma Cell-Derived Exosomal miR-21-5p Induces Macrophage M2 Polarization by Targeting RhoB. Int J Mol Sci.

[28] Hu Y, Li Y, Xiong H (2024). Exosomal SLC16A1-AS1-induced M2 macrophages polarization facilitates hepatocellular carcinoma progression. Int J Biol Sci.

[29] Snaebjornsson MT, Poeller P, Komkova D (2025). Targeting aldolase A in hepatocellular carcinoma leads to imbalanced glycolysis and energy stress due to uncontrolled FBP accumulation. Nat Metab.

[30] Lv W, Shi L, Pan J, Wang S (2022). Comprehensive prognostic and immunological analysis of CCT2 in pan-cancer. Front Oncol.

[31] Gordon SR, Maute RL, Dulken BW (2017). PD-1 expression by tumour-associated macrophages inhibits phagocytosis and tumour immunity. Nature.

[32] Yu J, Wei Z, Li Q (2021). Advanced Cancer Starvation Therapy by Simultaneous Deprivation of Lactate and Glucose Using a MOF Nanoplatform. Adv Sci.

[33] Chen X, Ma C, Li Y (2024). Trim21-mediated CCT2 ubiquitination suppresses malignant progression and promotes CD4+T cell activation in breast cancer. Cell Death Dis.

[34] Zhang J, Yu Z, Wang M (2025). Enhanced exosome secretion regulated by microglial P2X7R in the medullary dorsal horn contributes to pulpitis-induced pain. Cell Biosci.

[35] Liu J, Huang L, Zhu Y (2021). Exploring the Expression and Prognostic Value of the TCP1 Ring Complex in Hepatocellular Carcinoma and Overexpressing Its Subunit 5 Promotes HCC Tumorigenesis. Front Oncol.

[36] Yagita Y, Zavodszky E, Peak-Chew S-Y, Hegde RS (2023). Mechanism of orphan subunit recognition during assembly quality control. Cell.

[37] Sun J, He D, Fu Y (2021). A novel lncRNA ARST represses glioma progression by inhibiting ALDOA-mediated actin cytoskeleton integrity. J Exp Clin Cancer Res.

[38] Wan N, Wang N, Yu S (2022). Cyclic immonium ion of lactyllysine reveals widespread lactylation in the human proteome. Nat Methods.

[39] Rojas-Gómez A, Dosil SG, Chichón FJ (2023). Chaperonin CCT controls extracellular vesicle production and cell metabolism through kinesin dynamics. J Extracell Vesicles.

[40] Søndergaard JN, Sommerauer C, Atanasoai I (2022). CCT3-LINC00326 axis regulates hepatocarcinogenic lipid metabolism. Gut.

[41] Chen S, Tian Y, Ju A, Li B, Fu Y, Luo Y (2022). Suppression of CCT3 Inhibits Tumor Progression by Impairing ATP Production and Cytoplasmic Translation in Lung Adenocarcinoma. Int J Mol Sci.

[42] Fang J, Ma Y, Li Y (2022). CCT4 knockdown enhances the sensitivity of cisplatin by inhibiting glycolysis in human esophageal squamous cell carcinomas. Mol Carcinog.

[43] Chen J, Huang Z, Chen Y (2025). Lactate and lactylation in cancer. Signal Transduct Target Ther.

[44] Han S, Bao X, Zou Y (2023). d-lactate modulates M2 tumor-associated macrophages and remodels immunosuppressive tumor microenvironment for hepatocellular carcinoma. Sci Adv.

[45] Toledo B, Zhu Chen L, Paniagua-Sancho M, Marchal JA, Perán M, Giovannetti E (2024). Deciphering the performance of macrophages in tumour microenvironment: a call for precision immunotherapy. J Hematol Oncol.

[46] Colegio OR, Chu N-Q, Szabo AL (2014). Functional polarization of tumour-associated macrophages by tumour-derived lactic acid. Nature.

[47] Liu H, Liang Z, Cheng S (2023). Mutant KRAS Drives Immune Evasion by Sensitizing Cytotoxic T-Cells to Activation-Induced Cell Death in Colorectal Cancer. Adv Sci.

[48] Guo X, Tan S, Wang T (2023). NAD + salvage governs mitochondrial metabolism, invigorating natural killer cell antitumor immunity. Hepatology.

[49] Yang X, Lu Y, Hang J (2020). Lactate-Modulated Immunosuppression of Myeloid-Derived Suppressor Cells Contributes to the Radioresistance of Pancreatic Cancer. Cancer Immunol Res.

[50] Sun H, Zhang T, Zhang X (2025). Exosomal CCT6A Secreted by Cancer-Associated Fibroblasts Interacts with β-Catenin to Enhance Chemoresistance and Tumorigenesis in Gastric Cancer. Adv Sci.

[51] Cao X, Kong X, Zhou Y, Lan L, Luo L, Yin Z (2015). Glutathione S-transferase P1 suppresses iNOS protein stability in RAW264.7 macrophage-like cells after LPS stimulation. Free Radic Res.

[52] Dong X, Sun R, Wang J (2020). Glutathione S-transferases P1-mediated interleukin-6 in tumor-associated macrophages augments drug-resistance in MCF-7 breast cancer. Biochem Pharmacol.

[53] Bassiouni R, Nemec KN, Iketani A (2016). Chaperonin Containing TCP-1 Protein Level in Breast Cancer Cells Predicts Therapeutic Application of a Cytotoxic Peptide. Clin Cancer Res.

[54] Verma J, Subbarao N (2023). *In silico* identification of small molecule protein-protein interaction inhibitors: targeting hotspot regions at the interface of MXRA8 and CHIKV envelope protein. J Biomol Struct Dyn.

[55] Wang C, Zhang Y, Chen W, Wu Y, Xing D (2024). New-generation advanced PROTACs as potential therapeutic agents in cancer therapy. Mol Cancer.

[56] Ni X, Lu C-P, Xu G-Q, Ma J-J (2024). Transcriptional regulation and post-translational modifications in the glycolytic pathway for targeted cancer therapy. Acta Pharmacol Sin.

[57] Han Q-F, Li W-J, Hu K-S (2022). Exosome biogenesis: machinery, regulation, and therapeutic implications in cancer. Mol Cancer.

