# Supplementary material for: CCT2 Orchestrates Glycolysis and Exosome-Mediated M2 Macrophage Polarization in HCC tumorigenesis
Source: Int J Biol Sci. 2026 Mar 17;22(7):3389–410. doi: 10.7150/ijbs.122859 (PMC13085899; doi:10.7150/ijbs.122859)
Supplement: Supplementary file 1 — Supplementary figures and tables. [file ijbsv22p3389s1.pdf]

Supplementary data

1. Supplementary Tables

Supplementary Table 1

Primer sequences used in this study.

Primer name	Sequence (5'-3')
GAPDH-F	ACCACAGTCCATGCCATCAC
GAPDH-R	TCCACCACCCTGTTGCTGTA
CCT2-F	GAACGCCTAGCTCTTGTCACA
CCT2-R	GCACCACGCAAAACAATGGTA
CD206-F	CTACAAGGGATCGGGTTTATGGA
CD206-R	TTGGCATTGCCTAGTAGCGTA
IL10-F	TCAAGGCGCATGTGAACTCC
IL10-R	GATGTCAAACTCACTCATGGCT
TGFβ1-F	CAATTCCTGGCGATACCTCAG
TGFβ1-R	GCACAACTCCGGTGACATCAA
cct2-F	AAATTCTCCTAAGCAGTGGACG
cct2-R	CGGCTAAGACAGTAACAGAGGT
gapdh-F	TGACCTCAACTACATGGTCTACA
gapdh-R	CTTCCCATTCTCGGCCTTG
ALDOA-F	GCTGTCACTGGGATCACCTTC
ALDOA-R	GCTCGGAGTGTACTTTCCTTGA
CCT4-F	GTTCTGCTGAGTTAGCTGAGG
CCT4-R	TTCAGTTAATCGTAGGGCCAAC
CCT5-F	TGGAACTGTCCAAGTCTCAGG
CCT5-R	CATCGGCTATTCTGATTGGGTG
iNOS-F	TTCAGTATCACAACCTCAGCAAG
iNOS-R	TGGACCTGCAAGTTAAAATCCC
TNF-F	GAGGCCAAGCCCTGGTATG
TNF-R	CGGGCCGATTGATCTCAGC
IL6-F	CCTGAACCTTCCAAAGATGGC
IL6-R	TTCACCAGGCAAGTCTCCTCA
CD274-F	GGACAAGCAGTGACCATCAAG
CD274-R	CCCAGAATTACCAAGTGAGTCCT
CCL2-F	CAGCCAGATGCAATCAATGCC
CCL2-R	TGGAATCCTGAACCCACTTCT
CCL22-F	ATTACGTCCGTTACCGTCTGC
CCL22-R	TCCCTGAAGGTTAGCAACACC
CCL5-F	CCAGCAGTCGTCTTTGTCAC
CCL5-R	CTCTGGGTTGGCACACACTT
CXCL10-F	GTGGCATTCAAGGAGTACCTC
CXCL10-R	TGATGGCCTTCGATTCTGGATT

CXCL12-F	ATTCTCAACACTCCAAACTGTGC
CXCL12-R	ACTTTAGCTTCGGGTCAATGC
CXCL5-F	AGCTGCGTTGCGTTTGTTTAC
CXCL5-R	TGGCGAACACTTGCAGATTAC
CXCL8-F	ACTGAGAGTGATTGAGAGTGGAC
CXCL8-R	AACCCTCTGCACCCAGTTTTC
CXCL9-F	CCAGTAGTGAGAAAGGGTCGC
CXCL9-R	AGGGCTTGGGGCAAATTGTT
GSTP1-F	CCCTACACCGTGGTCTATTTCC
GSTP1-R	CAGGAGGCTTTGAGTGAGC
si-CCT2-1	TTATCGAGGAAGTCATGATTG
si-CCT2-2	CGGGCACAACATTATCCTCAA
si-ALDOA-1	CCATGCTTGCACTCAGAAGTT
si-ALDOA-2	TGGCGTTGTGTGCTGAAGATT
si-CCT4	CCCTATGTGTTATTCGTTGTT
si-CCT5	CGCGATAATCGTGTGGTGTAT
shCCT2-1-F	CCGGTTATCGAGGAAGTCATGATTGCTCGAGCAATCATGAC TTCCTCGATAATTTTTG
shCCT2-1-R	AATTCAAAAATTATCGAGGAAGTCATGATTGCTCGAGCAAT CATGACTTCCTCGATAA
shNC-F	CCGGTTCTCCGAACGTGTCACGTCTCGAGACGTGACACGTT CGGAGAATTTTTG
shNC-R	AATTCAAAAATTCTCCGAACGTGTCACGTCTCGAGACGTGA CACGTTCGGAGAA
shCCT2-2-F	CCGGCGGGCACAACATTATCCTCAACTCGAGTTGAGGATAA TGTTGTGCCCGTTTTTG
shCCT2-2-R	AATTCAAAAACGGGCACAACATTATCCTCAACTCGAGTTGA GGATAATGTTGTGCCCG
shALDOA-1-F	CCGGCCATGCTTGCACTCAGAAGTTCTCGAGAACTTCTGAG TGCAAGCATGGTTTTTG
shALDOA-1-R	AATTCAAAAACCATGCTTGCACTCAGAAGTTCTCGAGAACT TCTGAGTGCAAGCATGG
shALDOA-2-F	CCGGTGGCGTTGTGTGCTGAAGATTCTCGAGAATCTTCAGC ACACAACGCCATTTTTG
shALDOA-2-R	AATTCAAAAATGGCGTTGTGTGCTGAAGATTCTCGAGAATC TTCAGCACACAACGCCA
shGSTP1-1-F	CCGGCCTCACCCTGTACCAGTCCAACTCGAGTTGGACTGGT ACAGGGTGAGGTTTTTG
shGSTP1-1-R	AATTCAAAAACCTCACCCTGTACCAGTCCAACTCGAGTTGG ACTGGTACAGGGTGAGG
shGSTP1-2-F	CCGGCTGCAAATACATCTCCCTCATCTCGAGATGAGGGAGA TGTATTTGCAGTTTTTG
shGSTP1-2-R	AATTCAAAAACTGCAAATACATCTCCCTCATCTCGAGATGA GGGAGATGTATTTGCAG

shcct2-1-F	CCGGGCTGTAGCAATGGAGTCGTTTCTCGAGAAACGACTCCATTGCTACAGCTTTTTG
shcct2-1-R	AATTCAAAAAGCTGTAGCAATGGAGTCGTTTCTCGAGAAACGACTCCATTGCTACAGC
shcct2-2-F	CCGGGCTACCATTCTCAAGAACATTCTCGAGAATGTTCTTGAGAATGGTAGCTTTTTG
shcct2-2-R	AATTCAAAAAGCTACCATTCTCAAGAACATTCTCGAGAATGTTCTTGAGAATGGTAGC

Supplementary Table 2

Primary antibodies used in this study.

Antigens	Manufacturer	Number
CCT2	Abclonal	A6546
CCT2	Proteintech	68214-1-Ig
GAPDH	Abclonal	A19056
KI67	Proteintech	28074-1-AP
cct2	Proteintech	68214-1-Ig
ALDOA	Proteintech	11217-1-AP
ALDOA	Proteintech	67453-1-Ig
GST	Proteintech	66001-2-Ig
Flag	Proteintech	66008-4-Ig
HA	Proteintech	66006-2-Ig
ACTB	Proteintech	66009-1-Ig
UB	Proteintech	10201-2-AP
CCT4	Abcam	ab205013
CCT5	Proteintech	11603-1-AP
LC3B	Abclonal	A19665
CD206	Proteintech	18704-1-AP
CD68	Abclonal	A23205
CD206	Proteintech	18704-1-AP
F4/80	Proteintech	28463-1-AP
CD8-PerCP-Cy5.5	BD Pharmingen	561109
IFN-PE	Proteintech	PE-65153
PD-1-BV421	BD Pharmingen	562584
CD3-APC	Proteintech	APC-65077
CD45-APC-Cy7	BD Pharmingen	561037
FVS510	BD Pharmingen	564406
CD11B-BV510	BD Pharmingen	563088
CD68-PerCP	Abclonal	A26255
CD86-APC	Proteintech	APC-65165
CD206-PE	Proteintech	PE-65155
CD63	Abclonal	A5271
Calnexin	Proteintech	10427-2-AP
Alix	Proteintech	12422-1-AP
HSP70	Abclonal	A23457
GSTP1	Proteintech	66715-1-Ig
GSTP1	Abclonal	A19061

Supplementary Table 3

CCT2 expression in HCCDB

Dataset	*P*-value	Type	Nums	Mean	STD	IQR	Significance
HCCDB1	7.61E-07	HCC	100	10.94	0.3944	0.4073	***
		Adjacent	97	10.7	0.2584	0.2887	
HCCDB3	1.25E-07	HCC	268	3.448	1.288	1.358	***
		Adjacent	243	2.954	0.7377	0.733	
		Cirrhotic	40	3.969	0.6486	0.9761	
		Healthy	6	2.668	0.416	0.7394	
HCCDB4	1.13E-34	HCC	240	9.029	0.6992	0.7304	***
		Adjacent	193	8.279	0.45	0.5278	
HCCDB6	2.20E-48	HCC	225	8.229	0.7507	0.9005	***
		Adjacent	220	7.176	0.567	0.7133	
HCCDB7	0.001171	HCC	80	13.71	0.367	0.342	**
		Adjacent	82	13.53	0.3192	0.3548	
HCCDB11	0.001798	HCC	88	12.26	0.9223	0.8792	**
		Adjacent	48	11.69	1.016	0.9535	
HCCDB12	0.000003313	HCC	81	13.12	0.4843	0.606	***
		Adjacent	80	12.8	0.3539	0.474	
HCCDB13	0.0003584	HCC	228	8.297	0.7034	1.014	***
		Adjacent	168	8.073	0.5365	0.6779	
HCCDB15	3.14E-16	HCC	351	11.21	0.5167	0.65	***
		Adjacent	49	10.78	0.2469	0.35	
HCCDB18	4.90E-29	HCC	212	5.422	0.6951	0.725	***
		Adjacent	177	4.723	0.4147	0.44	
HCCDB20	0.01689	HCC	53	6.097	0.511	0.4269	*
		Adjacent	29	5.784	0.5681	0.6089	
		Cirrhotic	8	5.488	0.4325	0.7507	
		Healthy	6	5.603	0.6596	0.6384	
HCCDB22	0.0001863	HCC	64	10.78	0.7898	0.6792	***
		Adjacent	30	10.26	0.4822	0.6291	
HCCDB24	2.53E-10	HCC	115	10.13	0.4647	0.6814	***
		Adjacent	52	9.602	0.4407	0.7283	
HCCDB25	1.85E-27	HCC	158	11.43	0.5845	0.7119	***
		Adjacent	158	10.75	0.3951	0.435	
HCCDB27	3.49E-08	HCC	70	9.525	0.541	0.6737	***
		Adjacent	37	8.91	0.472	0.4012	
HCCDB29	0.003104	HCC	35	10.25	0.5178	0.661	**
		Adjacent	59	9.957	0.2481	0.3036	
		Normal	13	9.864	0.2923	0.1958	
HCCDB30	3.77E-10	HCC	54	4.243	0.5226	0.8628	***
		Adjacent	47	3.647	0.3127	0.3808	
		Normal	15	3.56	0.2889	0.2757	

Nums, number of samples; STD, standard deviation; IQR, interquartile range; NS, not significant, *$P < 0.05$, ** $P < 0.01$, *** $P < 0.001$.

Supplementary Table 4

CCT3 expression in HCCDB

Dataset	*P*-value	Type	Nums	Mean	STD	IQR	Significance
HCCDB1	1.53E-38	HCC	100	13.01	0.3759	0.5342	***
		Adjacent	97	12.26	0.191	0.248	
HCCDB3	6.01E-61	HCC	268	14.53	4.447	5.779	***
		Adjacent	243	8.63	1.342	1.515	
		Cirrhotic	40	8.181	1.415	1.79	
		Healthy	6	7.14	0.856	1.257	
HCCDB4	1.79E-108	HCC	240	9.415	0.5369	0.7412	***
		Adjacent	193	8.131	0.2013	0.2249	
HCCDB6	4.46E-107	HCC	225	9.586	0.6356	0.943	***
		Adjacent	220	8.017	0.3882	0.314	
HCCDB7	5.57E-21	HCC	80	14.55	0.224	0.2097	***
		Adjacent	82	14.19	0.1929	0.2562	
HCCDB11	0.006438	HCC	88	8.084	0.9996	1.682	**
		Adjacent	48	7.661	0.7547	1.141	
HCCDB12	1.30E-11	HCC	81	13.91	0.6542	0.876	***
		Adjacent	80	13.27	0.4261	0.3405	
HCCDB13	6.32E-79	HCC	228	10.54	0.5826	0.7582	***
		Adjacent	168	9.434	0.2984	0.3503	
HCCDB15	2.83E-64	HCC	351	13.19	0.6453	0.8	***
		Adjacent	49	12.09	0.2041	0.26	
HCCDB18	2.85E-86	HCC	212	6.868	0.6879	0.99	***
		Adjacent	177	5.392	0.3137	0.31	
HCCDB20	0.008197	HCC	53	5.488	0.571	0.5656	**
		Adjacent	29	5.188	0.4181	0.6558	
		Cirrhotic	8	5.563	0.3717	0.3878	
		Healthy	6	5.56	0.234	0.4124	
HCCDB22	7.82E-23	HCC	64	11.11	0.6542	0.7248	***
		Adjacent	30	9.755	0.3377	0.6184	
HCCDB24	6.17E-32	HCC	115	10.84	0.5056	0.7749	***
		Adjacent	52	9.943	0.2716	0.3945	
HCCDB25	6.22E-64	HCC	158	12.77	0.6608	0.7358	***
		Adjacent	158	11.37	0.3051	0.3453	
HCCDB27	4.23E-19	HCC	70	11.3	0.5758	0.7553	***
		Adjacent	37	10.26	0.3847	0.3745	
HCCDB29	0.00000209	HCC	35	9.506	0.5428	0.7415	***
		Adjacent	59	8.973	0.244	0.2766	
		Normal	13	8.69	0.1192	0.1839	
HCCDB30	1.60E-13	HCC	54	5.492	0.7075	0.9408	***
		Adjacent	47	4.528	0.3395	0.3274	
		Normal	15	4.234	0.1347	0.1919	

Nums, number of samples; STD, standard deviation; IQR, interquartile range; NS, not significant, *$P < 0.05$, ** $P < 0.01$, *** $P < 0.001$.

Supplementary Table 5

CCT5 expression in HCCDB

Dataset	*P*-value	Type	Nums	Mean	STD	IQR	Significance
HCCDB1	8.12E-08	HCC	100	10.38	0.4934	0.6375	***
		Adjacent	97	10.05	0.3102	0.4081	
HCCDB3	1.52E-19	HCC	268	6.133	2.097	2.925	***
		Adjacent	243	4.76	1.026	1.117	
		Cirrhotic	40	5.126	1.161	1.247	
		Healthy	6	4.098	0.5916	0.9788	
HCCDB4	4.02E-09	HCC	240	7.33	0.4437	0.5808	***
		Adjacent	193	7.121	0.2728	0.3305	
HCCDB6	3.23E-72	HCC	225	8.456	0.6264	0.825	***
		Adjacent	220	7.341	0.3846	0.3877	
HCCDB7	2.79E-09	HCC	80	14.27	0.4292	0.459	***
		Adjacent	82	13.91	0.2721	0.2998	
HCCDB11	0.01624	HCC	88	7.965	1.247	1.643	*
		Adjacent	48	8.625	1.617	2.611	
HCCDB12	4.78E-07	HCC	81	9.239	0.5653	0.82	***
		Adjacent	80	8.809	0.4692	0.6609	
HCCDB13	7.03E-12	HCC	228	8.44	0.8546	1.159	***
		Adjacent	168	7.958	0.4885	0.557	
HCCDB15	5.65E-37	HCC	351	11.92	0.6165	0.77	***
		Adjacent	49	11.25	0.193	0.25	
HCCDB18	2.07E-51	HCC	212	5.192	0.6685	0.83	***
		Adjacent	177	4.218	0.3843	0.38	
HCCDB20	0.07755	HCC	53	4.374	0.5492	0.5699	NS
		Adjacent	29	4.197	0.3433	0.6153	
		Cirrhotic	8	4.141	0.3164	0.4702	
		Healthy	6	4.067	0.5936	0.8547	
HCCDB22	0.03501	HCC	64	7.422	0.5908	0.9363	*
		Adjacent	30	7.226	0.2964	0.3105	
HCCDB24	0.06481	HCC	115	8.288	0.473	0.6372	NS
		Adjacent	52	8.402	0.3054	0.3879	
HCCDB25	2.35E-42	HCC	158	11.66	0.6523	0.8843	***
		Adjacent	158	10.68	0.3328	0.4852	
HCCDB27	5.52E-08	HCC	70	8.412	0.5216	0.7622	***
		Adjacent	37	7.952	0.2919	0.371	
HCCDB29	0.2915	HCC	35	6.973	0.3473	0.4705	NS
		Adjacent	59	6.902	0.2332	0.3279	
		Normal	13	6.901	0.2309	0.2637	
HCCDB30	3.16E-07	HCC	54	3.804	0.6364	0.8992	***
		Adjacent	47	3.254	0.3233	0.2879	
		Normal	15	3.002	0.1709	0.2691	

Nums, number of samples; STD, standard deviation; IQR, interquartile range; NS, not significant, **P* < 0.05, ** *P* < 0.01, *** *P* < 0.001.

Supplementary Table 6

CCT6A expression in HCCDB

Dataset	*P*-value	Type	Nums	Mean	STD	IQR	Significance
HCCDB1	1.17E-17	HCC	100	11.18	0.4025	0.4698	***
		Adjacent	97	10.71	0.286	0.3315	
HCCDB3	1.69E-21	HCC	268	4.271	1.278	1.601	***
		Adjacent	243	3.341	0.7794	0.99	
		Cirrhotic	40	3.97	0.6619	0.802	
		Healthy	6	3.244	0.7499	1.406	
HCCDB4	1.31E-53	HCC	240	7.853	0.5243	0.8164	***
		Adjacent	193	7.156	0.2278	0.2902	
HCCDB6	1.31E-84	HCC	225	8.318	0.6658	0.852	***
		Adjacent	220	6.787	0.6494	0.6873	
HCCDB7	3.58E-09	HCC	80	12.31	0.3783	0.5048	***
		Adjacent	82	11.97	0.326	0.4565	
HCCDB11	0.6663	HCC	88	7.291	0.6965	0.7759	NS
		Adjacent	48	7.23	0.8157	0.8846	
HCCDB12	3.08E-09	HCC	81	11.66	0.5499	0.7785	***
		Adjacent	80	11.2	0.3642	0.3852	
HCCDB13	2.18E-20	HCC	228	9.571	0.4611	0.6318	***
		Adjacent	168	9.159	0.3749	0.4305	
HCCDB15	2.78E-34	HCC	351	11.87	0.5354	0.7	***
		Adjacent	49	11.04	0.2628	0.26	
HCCDB18	5.99E-67	HCC	212	5.672	0.595	0.695	***
		Adjacent	177	4.582	0.403	0.45	
HCCDB20	0.0004246	HCC	53	5.246	0.5473	0.6078	**
		Adjacent	29	4.843	0.425	0.5089	
		Cirrhotic	8	4.7	0.2944	0.3459	
		Healthy	6	4.675	0.702	1.107	
HCCDB22	0.008824	HCC	64	7.355	0.723	1.202	**
		Adjacent	30	7.66	0.3813	0.5569	
HCCDB24	2.96E-17	HCC	115	8.95	0.3742	0.562	***
		Adjacent	52	8.425	0.2911	0.3034	
HCCDB25	1.27E-54	HCC	158	11.81	0.6245	0.9264	***
		Adjacent	158	10.64	0.4069	0.5209	
HCCDB27	8.03E-13	HCC	70	9.662	0.5108	0.7229	***
		Adjacent	37	8.935	0.3822	0.3707	
HCCDB29	0.5694	HCC	35	8.22	0.3812	0.5272	NS
		Adjacent	59	8.179	0.2389	0.3395	
		Normal	13	7.983	0.1352	0.09676	
HCCDB30	2.10E-11	HCC	54	4.953	0.6825	0.8583	***
		Adjacent	47	4.12	0.3766	0.3966	
		Normal	15	3.874	0.297	0.4259	

Nums, number of samples; STD, standard deviation; IQR, interquartile range; NS, not significant, $*P < 0.05$, $** P < 0.01$, $*** P < 0.001$.

Supplementary Table 7

Relationship between CCT2 expression and clinicopathologic characteristics of TCGA-LIHC patients.

	**Total** *n = 341*	**CCT2_High** *n = 172*	**CCT2_Low** *n = 169*	***p.value***
**Age:**				0.125
<50	69 (20.2%)	41 (23.8%)	28 (16.6%)	
≥50	272 (79.8%)	131 (76.2%)	141 (83.4%)	
**Gender:**				0.015
Female	107 (31.4%)	43 (25.0%)	64 (37.9%)	
Male	234 (68.6%)	129 (75.0%)	105 (62.1%)	
**Status:**				0.066
Alive	227 (66.6%)	106 (61.6%)	121 (71.6%)	
Dead	114 (33.4%)	66 (38.4%)	48 (28.4%)	
**Pathologic stage:**				0.034
Stage I+II	252 (73.9%)	118 (68.6%)	134 (79.3%)	
Stage III+IV	89 (26.1%)	54 (31.4%)	35 (20.7%)	
**Invasion depth:**				0.031
T1+T2	256 (75.1%)	120 (69.8%)	136 (80.5%)	
T3+T4	85 (24.9%)	52 (30.2%)	33 (19.5%)	

Supplementary Table 8

Baseline information on clinical samples from 23 patients with HCC included in this study.

	**CCT2_Low (*n* = 11)**	**CCT2_High (*n* = 12)**	**Total (*n* = 23)**
**Age:**			
Mean (SD)	55.1 (6.50)	53.8 (9.05)	54.4 (7.79)
Median [Min, Max]	54.0 [46.0, 70.0]	54.5 [38.0, 71.0]	54.0 [38.0, 71.0]
**Sex:**			
Male	8 (72.7%)	9 (75.0%)	17 (73.9%)
Female	3 (27.3%)	3 (25.0%)	6 (26.1%)
**HBV:**			
Negative	6 (54.5%)	4 (33.3%)	10 (43.5%)
Positive	5 (45.5%)	8 (66.7%)	13 (56.5%)
**HCV:**			
Negative	9 (81.8%)	12 (100%)	21 (91.3%)
Positive	2 (18.2%)	0 (0%)	2 (8.7%)
**MAFLD:**			
Absent	8 (72.7%)	8 (66.7%)	16 (69.6%)
Present	3 (27.3%)	4 (33.3%)	7 (30.4%)
**BMI:**			
Mean (SD)	24.5 (3.97)	22.6 (3.21)	23.5 (3.64)
Median [Min, Max]	25.2 [19.8, 31.9]	22.2 [19.1, 28.5]	23.1 [19.1, 31.9]
**Alcohol_history:**			
No alcohol	1 (9.1%)	0 (0%)	1 (4.3%)
>0 drink	10 (90.9%)	12 (100%)	22 (95.7%)

2. Supplementary Figures

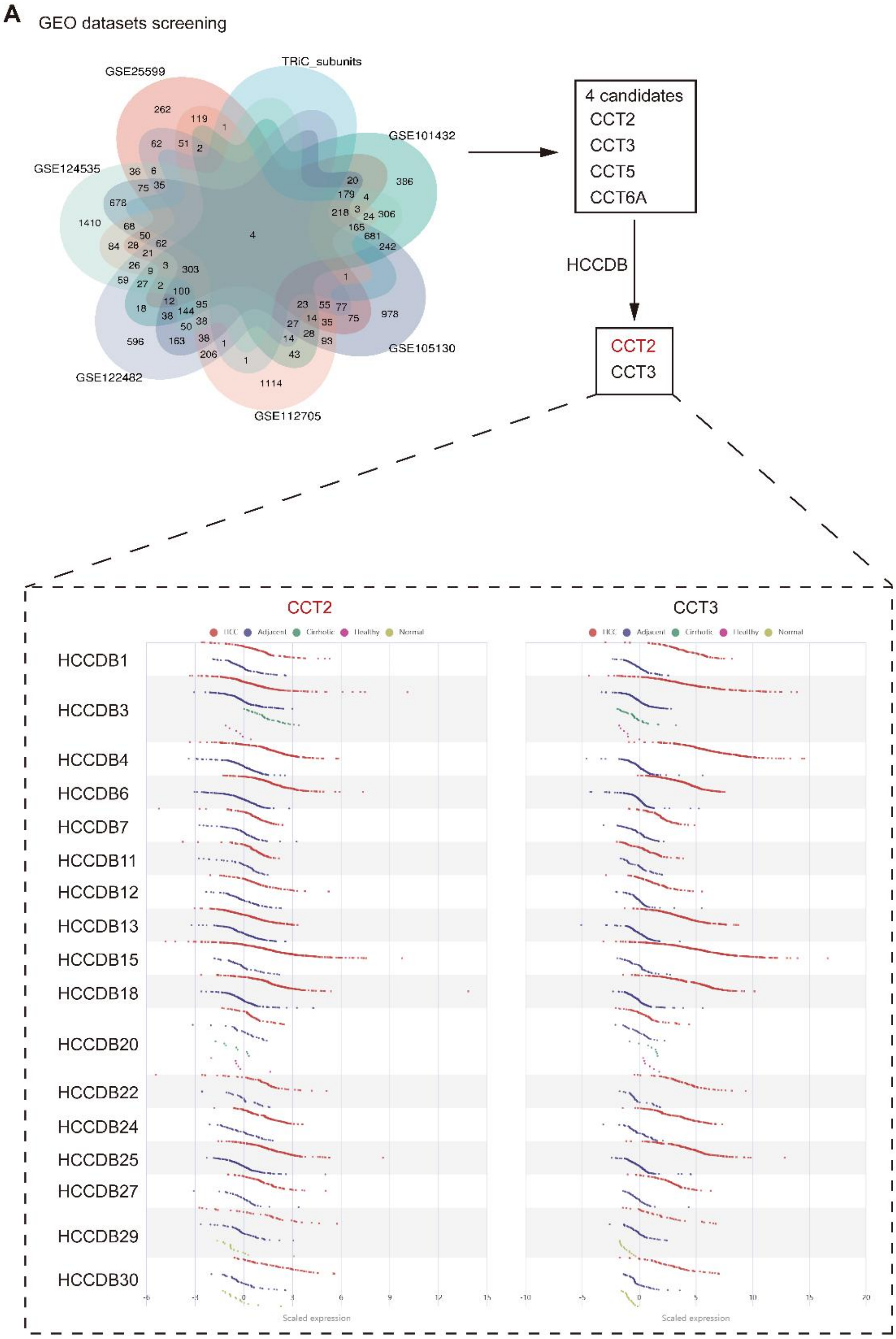

Supplementary Fig. 1. Integrated screening and validation of oncogenic TRiC subunits in HCC.

(A) Differentially expressed genes (tumor vs. adjacent normal; $P < 0.05$, $|\log2FC| > \log2(1.5)$) were identified across six GEO HCC datasets and intersected to obtain consistently dysregulated genes, which were further overlapped with known TRiC subunits, yielding four candidates (CCT2, CCT3, CCT5, and CCT6A).Expression patterns of these candidates were then evaluated across multiple independent cohorts in the HCCDB database, showing that only CCT2 and CCT3 are consistently upregulated in HCC tissues, with CCT2 prioritized for further study due to its potential role in HCC progression.

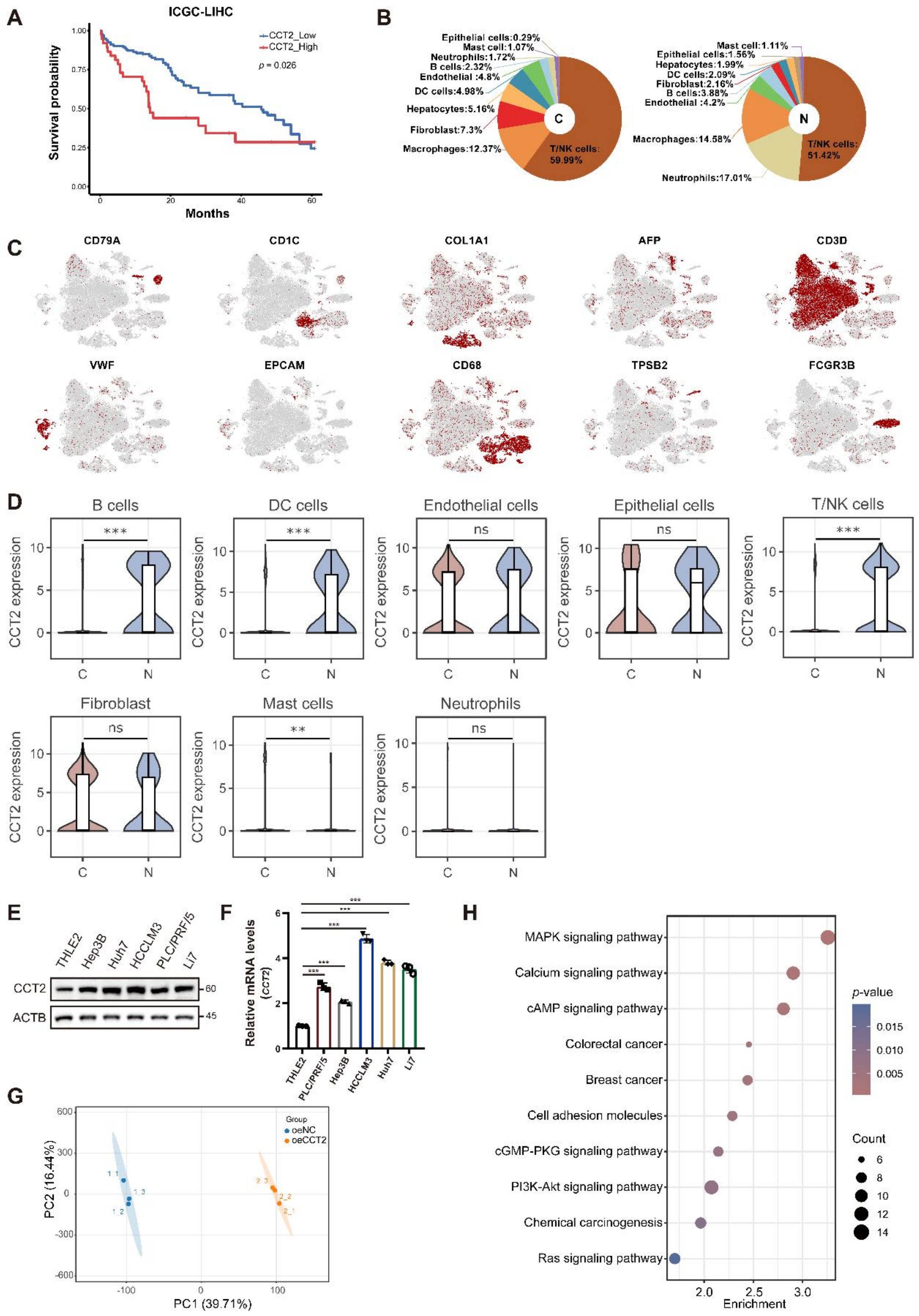
A
ICGC-LIHC
CCT2_Low
CCT2_High
p = 0.026
Survival probability
Months
B
Epithelial cells:0.29%
Mast cell:1.07%
Neutrophils:1.72%
B cells:2.32%
Endothelial :4.8%
DC cells:4.98%
Hepatocytes:5.16%
Fibroblast:7.3%
Macrophages:12.37%
T/NK cells: 59.99%
C
Mast cell:1.11%
Epithelial cells:1.56%
Hepatocytes:1.99%
DC cells:2.09%
Fibroblast:2.16%
B cells:3.88%
Endothelial :4.2%
Macrophages:14.58%
Neutrophils:17.01%
T/NK cells: 51.42%
N
C
CD79A
CD1C
COL1A1
AFP
CD3D
VWF
EPCAM
CD68
TPSB2
FCGR3B
D
B cells
DC cells
Endothelial cells
Epithelial cells
T/NK cells
Fibroblast
Mast cells
Neutrophils
CCT2 expression
E
THLE2
Hep3B
Huh7
HCCLM3
PLC/PRF/5
Li7
CCT2
ACTB
F
Relative mRNA levels (CCT2)
H
MAPK signaling pathway
Calcium signaling pathway
cAMP signaling pathway
Colorectal cancer
Breast cancer
Cell adhesion molecules
cGMP-PKG signaling pathway
PI3K-Akt signaling pathway
Chemical carcinogenesis
Ras signaling pathway
Enrichment
p-value
Count
G
Group
oeNC
oeCCT2
PC2 (16.44%)
PC1 (39.71%)

Supplementary Fig. 2. CCT2 is essential in HCC tumorigenesis and indicates poor prognosis. (A) Kaplan-Meier curves for overall survival (OS) stratified by CCT2 mRNA expression, analyzed using data retrieved from the ICGC database. (B) Proportions of different cell types in patient cancerous (C) and normal (N) tissues, as determined by scRNA-seq (n = 4). (C) t-SNE plots depicting the expression of the indicated cell markers in scRNA-seq data from HCC and adjacent normal tissues (n = 4). (D) CCT2 expression in the indicated cell populations of cancerous (C) and normal (N) tissues, analyzed from scRNA-seq data (n = 4). (E) Protein expression of CCT2 in THLE2 normal liver cell line and the indicated HCC cell lines analyzed by western blot analysis. (F) mRNA expression of CCT2 in THLE2 normal liver cell line and the indicated HCC cell lines analyzed by qPCR (n = 3). (G) Principal component analysis (PCA) of RNA-seq data from Huh7 cells transfected with oeNC and oeCCT2. (H) KEGG pathway enrichment analysis of differentially expressed genes in TCGA-LIHC, stratified by CCT2 expression. Differences were analyzed by Student's t-test or ANOVA. After ANOVA, Tukey's post-hoc test was used for multiple comparisons. Data are presented as mean ± SD. *$P < 0.05$, ** $P < 0.01$, *** $P < 0.001$.

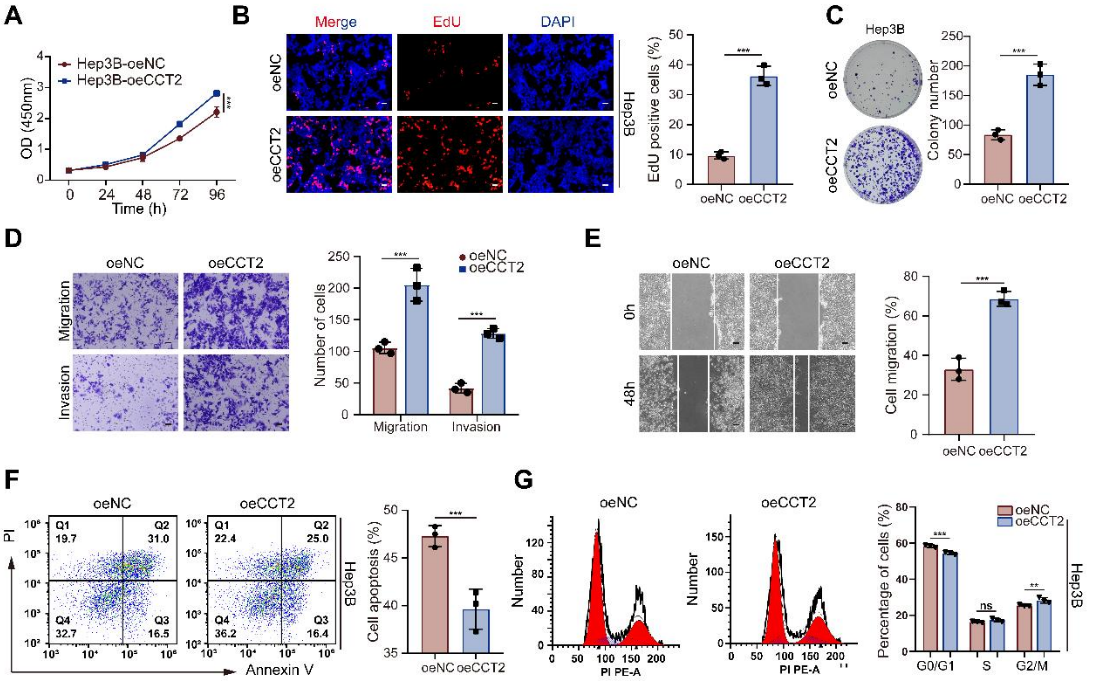

Supplementary Fig. 3. CCT2 contributes to HCC tumorigenesis and progression.

(A) CCK-8 assay for cell viability assessment of oeNC and oeCCT2 Hep3B cells (n = 5). (B) EdU assay for cell proliferation assessment of oeNC- and oeCCT2-transfected Hep3B cells (n = 3; scale bar, 100 μm). (C) Clonogenic assay for colony formation ability assessment of oeNC- and oeCCT2-transfected Hep3B cells (n = 3). (D) Transwell assay for the assessment of invasion and migration abilities of oeNC- and oeCCT2-transfected Hep3B cells (n = 3; scale bar, 100 μm). (E) Wound healing assay for migration assessment of oeNC- and oeCCT2-transfected Hep3B cells (n = 3; scale bar, 100 μm). (F) Flow cytometry for apoptosis analysis of oeNC- and oeCCT2-transfected Hep3B cells (n = 3). (G) Flow cytometry for cell cycle analysis of oeNC- and oeCCT2-transfected Hep3B (n = 3). Differences were analyzed by Student's t-test or ANOVA. After ANOVA, Tukey's post-hoc test was used for multiple comparisons. Three biological replicates were performed for cell experiments. Data are presented as mean ± SD. ns, not significant, *$P < 0.05$, ** $P < 0.01$, *** $P < 0.001$.

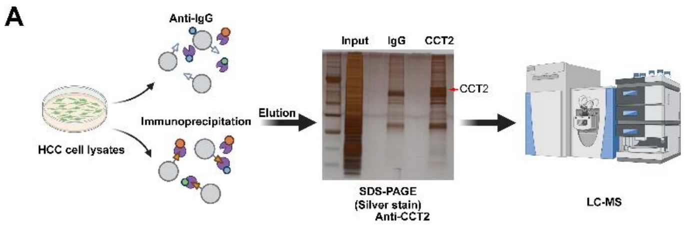

Supplementary Fig. 4. CCT2 promotes glycolysis in HCC cells via interaction with ALDOA.
(A) Schematic diagram of co-IP/MS for detecting CCT2 interactors in Huh7 cells.

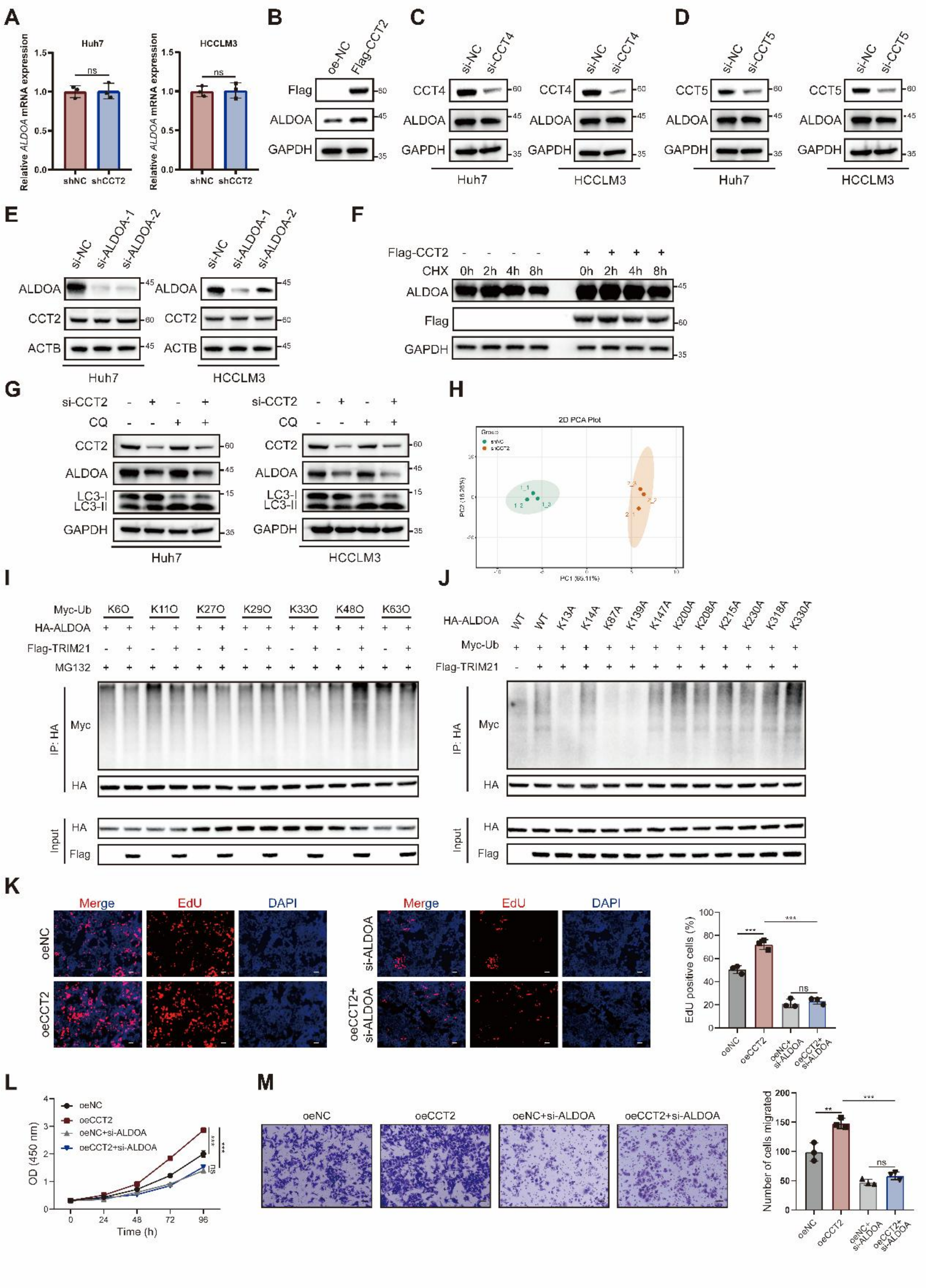

A
Huh7
HCCLM3
Relative ALDOA mRNA expression
ns
shNC
shCCT2
B
oe-NC
Flag-CCT2
Flag
ALDOA
GAPDH
C
si-NC
si-CCT4
CCT4
ALDOA
GAPDH
Huh7
HCCLM3
D
si-NC
si-CCT5
CCT5
ALDOA
GAPDH
Huh7
HCCLM3
E
si-NC
si-ALDOA-1
si-ALDOA-2
ALDOA
CCT2
ACTB
Huh7
HCCLM3
F
Flag-CCT2
CHX
0h
2h
4h
8h
ALDOA
Flag
GAPDH
G
si-CCT2
CQ
CCT2
ALDOA
LC3-I
LC3-II
GAPDH
Huh7
HCCLM3
H
2D PCA Plot
I
Myc-Ub
K6O
K11O
K27O
K29O
K33O
K48O
K63O
HA-ALDOA
Flag-TRIM21
MG132
IP: HA
Myc
HA
Input
HA
Flag
J
HA-ALDOA
WT
K13A
K14A
K87A
K139A
K147A
K200A
K208A
K215A
K230A
K318A
K330A
Myc-Ub
Flag-TRIM21
IP: HA
Myc
HA
Input
HA
Flag
K
Merge
EdU
DAPI
oeNC
oeCCT2
si-ALDOA
oeCCT2+ si-ALDOA
EdU positive cells (%)
oeNC
oeCCT2
oeNC+ si-ALDOA
oeCCT2+ si-ALDOA
ns
L
oeNC
oeCCT2
oeNC+si-ALDOA
oeCCT2+si-ALDOA
OD (450 nm)
Time (h)
ns
M
oeNC
oeCCT2
oeNC+si-ALDOA
oeCCT2+si-ALDOA
Number of cells migrated
ns

Supplementary Fig. 5. CCT2 enhances HCC glycolysis and tumorigenesis via ALDOA stabilization.

(A) mRNA expression of CCT2 and ALDOA in the shNC- and shCCT2-transfected Huh7 and HCCLM3 cells, determined by qPCR (n = 3). (B) Protein expression of Flag-CCT2 and ALDOA in Hep3B cells transfected with oeNC or Flag-CCT2, determined by western blot analysis. (C) Protein expression of ALDOA in Huh7 and HCCLM3 cells transfected with si-NC or si-CCT4, determined by western blot analysis. (D) Protein expression of ALDOA in Huh7 and HCCLM3 cells transfected with si-NC or si-CCT5, determined by western blot analysis. (E) Protein expression of CCT2 and ALDOA in the Huh7 and HCCLM3 cells, transfected with si-NC, si-ALDOA-1 or si-ALDOA-2, determined by western blot analysis. (F) CHX (200 μg/mL) chase assay to assess ALDOA degradation, determined by western blot analysis in Hep3B cells transfected with oeNC or Flag-CCT2. (G) Protein expression of CCT2 and ALDOA in Huh7 and HCCLM3 cells transfected with si-NC and si-CCT2-1, treated with or without chloroquine (CQ, 20 μM), determined by western blot analysis. (H) PCA analysis of mass spectrometry data for targeted metabolites in shNC- and shCCT2-transfected Huh7 cells (n = 3). (I) In vivo ubiquitination assay using ubiquitin linkage–specific mutants showing that TRIM21 predominantly induces K48-linked polyubiquitination of ALDOA in the presence of MG132. (J) Site-specific ubiquitination analysis using ALDOA lysine mutants identifying K13, K87, and K139 as major ubiquitination sites mediated by TRIM21. (K) EdU assay for cell proliferation assessment of oeNC- and oeCCT2-transfected Hep3B cells with or without si-ALDOA treatment (n = 3; scale bar, 50 μm). (L) CCK8 assay of oeNC- and oeCCT2-transfected Hep3B cells with or without si-ALDOA treatment (n = 3). (M) Transwell migration assay of oeNC- and oeCCT2-transfected Hep3B cells with or without si-ALDOA treatment (n = 3; scale bar, 100 μm). Differences were analyzed by Student's t-test. Data are presented as mean ± SD. CHX, cycloheximide; CQ, chloroquine. ns, not significant, *$P < 0.05$, ** $P < 0.01$, *** $P < 0.001$.

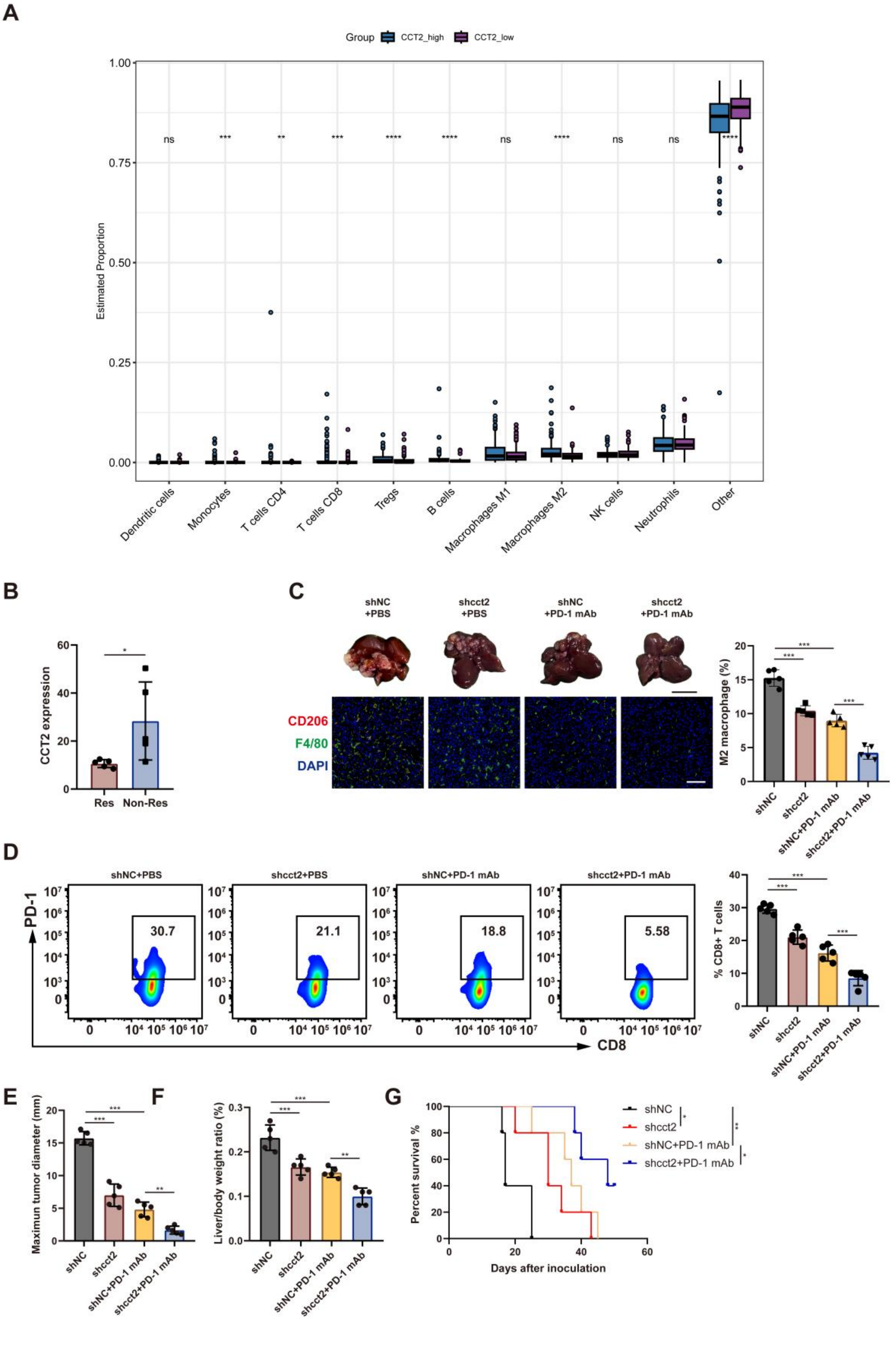

A
Group CCT2_high CCT2_low
Estimated Proportion
ns *** ** *** **** **** ns **** ns ns ****
Dendritic cells
Monocytes
T cells CD4
T cells CD8
Tregs
B cells
Macrophages M1
Macrophages M2
NK cells
Neutrophils
Other
B
CCT2 expression
Res
Non-Res
C
shNC +PBS
shcct2 +PBS
shNC +PD-1 mAb
shcct2 +PD-1 mAb
CD206
F4/80
DAPI
M2 macrophage (%)
shNC
shcct2
shNC+PD-1 mAb
shcct2+PD-1 mAb
D
shNC+PBS
shcct2+PBS
shNC+PD-1 mAb
shcct2+PD-1 mAb
PD-1
CD8
30.7
21.1
18.8
5.58
% CD8+ T cells
E
Maximun tumor diameter (mm)
F
Liver/body weight ratio (%)
G
Percent survival %
Days after inoculation
shNC
shcct2
shNC+PD-1 mAb
shcct2+PD-1 mAb

Supplementary Fig. 6. CCT2 promotes M2-like TAM infiltration in the TME and suppresses the efficacy of PD-1 mAb blockade.

(A) Immune infiltration analysis of TCGA-LIHC data based on CCT2 expression using the Quantiseq algorithm. (B) CCT2 expression in responders (Res) and non-responders (Non-Res) to anti–PD-1 therapy in the GSE215011 cohort. (C) Representative images of orthotopic liver tumors and immunofluorescence staining of tumor sections showing M2 macrophages (CD206, red), total macrophages (F4/80, green) (Scale bar, 50 μm), with quantification of M2 macrophage proportion in each group. (D) Flow cytometric analysis of PD-1^{+}CD8^{+} T cells in tumor tissues from indicated groups, with quantification shown on the right. (E) Maximum tumor diameter measured in mice from each treatment group. (F) Liver-to-body weight ratio of tumor-bearing mice in each group. (G) Kaplan–Meier survival curves of mice bearing orthotopic HCC tumors with indicated treatments. Data are presented as mean ± SD. ns, not significant, *$P < 0.05$, ** $P < 0.01$, *** $P < 0.001$.

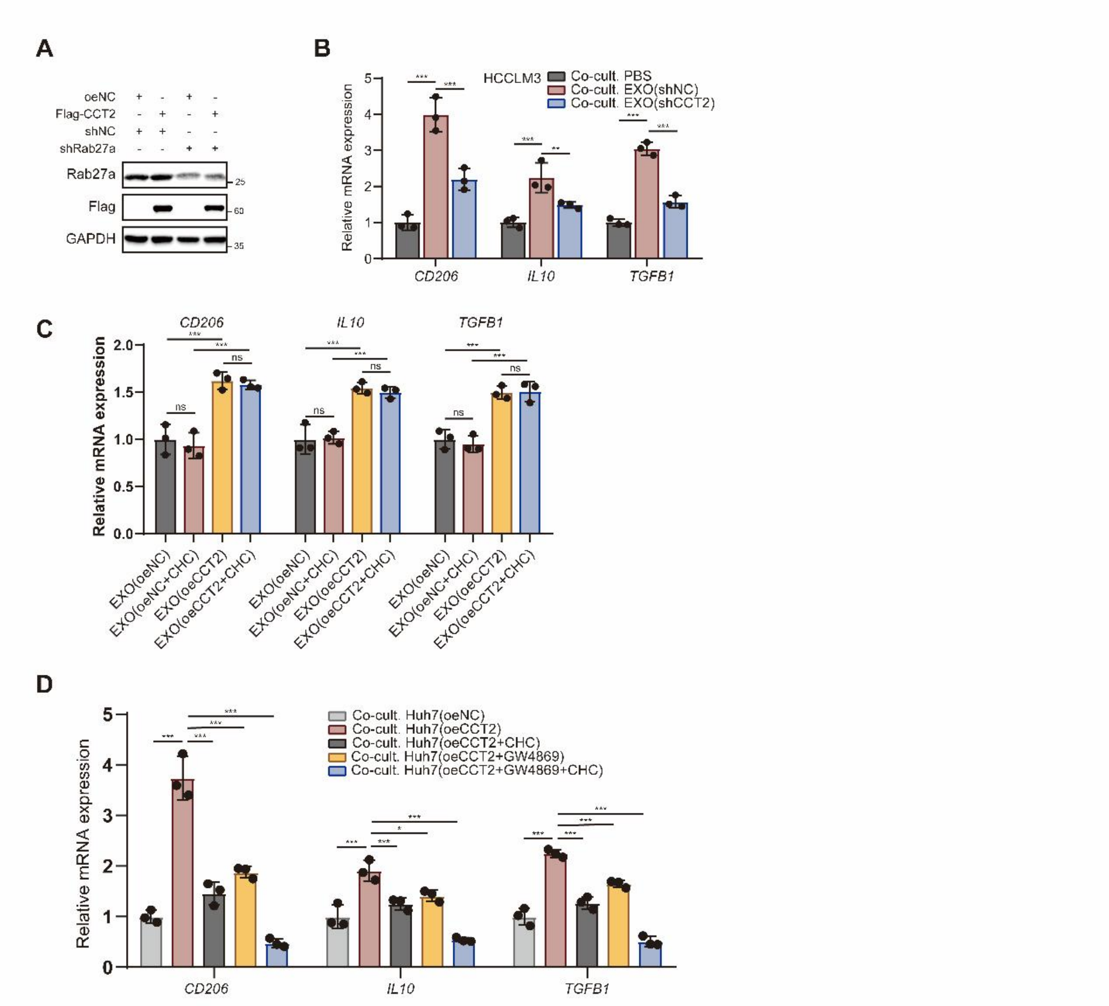

Supplementary Fig. 7. Both exosomes and lactate contribute to CCT2-mediated M2 polarization.

(A) Western blot analysis confirming Rab27a knockdown and Flag-CCT2 overexpression in Huh7 cells under the indicated conditions. (B) mRNA expression levels of *CD206*, *IL10*, and *TGFB1* in PMA-treated THP-1 cells directly treated with PBS or exosomes derived from HCCLM3 cells, determined by qPCR (n = 3). (C) mRNA expression levels of *CD206*, *IL10*, and *TGFB1* in PMA-treated THP-1 cells treated with exosomes isolated from oeNC- or oeCCT2-transfected HCC cells with or without CHC pretreatment, determined by qPCR (n = 3). (D) mRNA expression levels of *CD206*, *IL10*, and *TGFB1* in PMA-treated THP-1 cells co-cultured with oeNC- or oeCCT2-transfected Huh7 cells under the indicated conditions, including CHC treatment and/or GW4869-mediated inhibition of exosome release, determined by qPCR (n = 3). Differences were analyzed by Student's t-test or ANOVA. After ANOVA, Tukey's post-hoc test was used for multiple comparisons. Three biological replicates were performed for cell experiments. Data are presented as mean ± SD. Co-cult., co-culture with; CHC, chlorohydroxycinnamate. ns, not significant, $*P < 0.05$, $** P < 0.01$, $*** P < 0.001$.

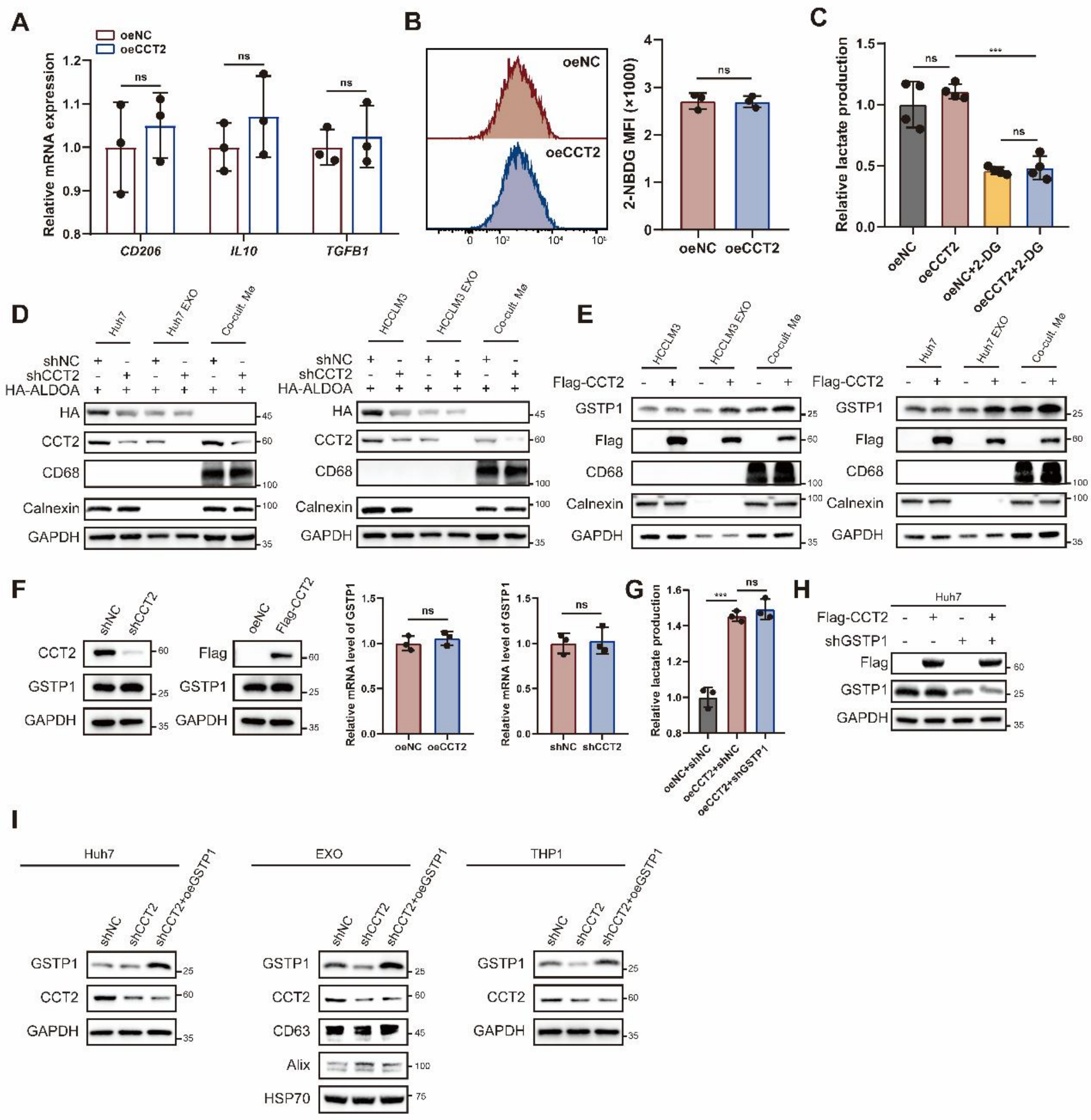

A
oeNC
oeCCT2
Relative mRNA expression
ns
CD206
IL10
TGFB1
B
oeNC
oeCCT2
2-NBDG MFI (×1000)
ns
C
Relative lactate production
ns
***
oeNC
oeCCT2
oeNC+2-DG
oeCCT2+2-DG
D
Huh7
Huh7 EXO
Co-cult. Mφ
shNC
shCCT2
HA-ALDOA
HA
CCT2
CD68
Calnexin
GAPDH
HCCLM3
HCCLM3 EXO
E
Flag-CCT2
GSTP1
Flag
F
shNC
shCCT2
oeNC
Flag-CCT2
Relative mRNA level of GSTP1
G
oeNC+shNC
oeCCT2+shNC
oeCCT2+shGSTP1
H
shGSTP1
I
EXO
THP1
shCCT2+oeGSTP1
CD63
Alix
HSP70

Supplementary Fig. 8. CCT2-mediated GSTP1 Transfer via Exosomes Promotes M2 Polarization.

(A) *CD206*, *IL10*, and *TGFB1* mRNA expression in PMA-treated THP1 cells transfected with oeNC or oeCCT2, determined by qPCR (n = 3). (B) Flow cytometric analysis of glucose uptake in PMA-treated THP-1 cells transfected with oeNC or oeCCT2 using 2-NBDG, with quantification of mean fluorescence intensity (MFI). (C) Lactate levels in culture supernatants of PMA-treated THP-1 cells transfected with oeNC or oeCCT2, with or without 2-DG (10 mM) treatment (n = 3). (D) Western blot analysis detecting HA-ALDOA, macrophage marker CD68, and Calnexin in Huh7 and HCCLM3 cells, their corresponding exosomes (Huh7/HCCLM3 EXO), and PMA-treated THP-1 cells co-cultured with the corresponding exosomes (Co-cult. Mø). (E) Western blot analysis of GSTP1 and Flag-CCT2 in HCC cells, their derived exosomes, and recipient macrophages after exosome co-culture. (F) Protein and mRNA expression levels of GSTP1 in HCC cells with CCT2 knockdown or overexpression, determined by western blot and qPCR. (G) Lactate levels in culture supernatants of oeNC, oeCCT2, and oeCCT2 + shGSTP1 Huh7 cells (n = 3). (H) Protein levels of Flag-CCT2 and GSTP1 in the indicated cells. (I) Western blot analysis of GSTP1 and CCT2 in donor HCC cells, derived exosomes, and recipient THP-1 cells after exosome co-culture under the indicated conditions. Differences were analyzed by Student's t-test. Three biological replicates were performed for cell experiments. Data are presented as mean ± SD. Co-cult., co-culture with; EXO, exosomes. ns, not significant, $*P < 0.05$, $** P < 0.01$, $*** P < 0.001$.

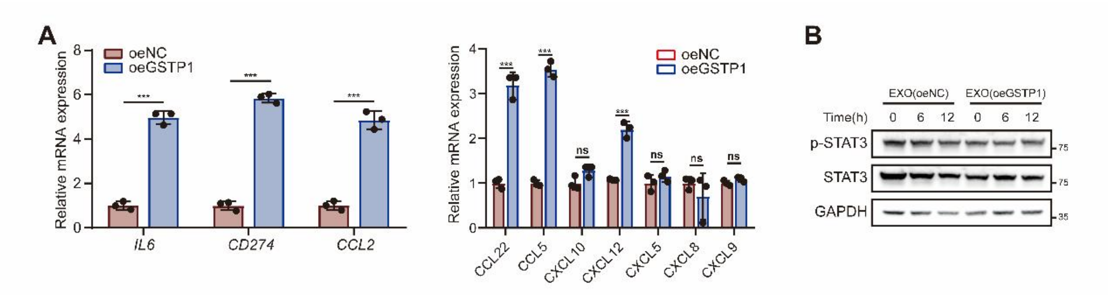

Supplementary Fig. 9. Exosomal GSTP1 activates the AKT–NF-κB signaling axis to promote M2 polarization.

(A) mRNA expression levels of IL6, CD274, CCL2 and other indicated immunosuppressive chemokines in PMA-treated THP-1 cells transduced with oeNC and oeGSTP1 vectors, determined by qPCR (n = 3). (B) Time-course western blot analysis of STAT3 phosphorylation in PMA-treated THP-1 cells treated with exosomes derived from oeNC- or oeGSTP1-transfected HCC cells, showing no significant change in p-STAT3 levels. Differences were analyzed by Student's t-test or ANOVA. After ANOVA, Tukey's post-hoc test was used for multiple comparisons. Three biological replicates were performed for cell experiments. Data are presented as mean ± SD. EXO, exosomes. ns, not significant, *$P < 0.05$, ** $P < 0.01$, *** $P < 0.001$.

3. Supplementary materials and methods

3.1 Transcriptome Sequencing

cDNA libraries were constructed, ligated with Illumina adapters, and amplified. Library quality was assessed and quantified, and sequencing was performed on an Illumina platform. Raw data were processed, trimmed, and aligned to the reference genome using STAR. Gene expression was quantified using HTSeq or featureCounts, and differential expression was analyzed with DESeq2.

3.2 Cell Proliferation Assays

Cell proliferation was assessed using colony formation, Cell Counting Kit-8 (CCK-8), and EdU staining assays. For the colony formation assay, 1,000–2,000 cells were seeded in 6-well plates and cultured for 2-3 weeks. Colonies were fixed with 4% paraformaldehyde (PFA), stained with 0.1% crystal violet, and counted under a light microscope. In the CCK-8 assay, 5×10^3 cells per well were seeded in 96-well plates. After 2 hours of incubation with 10 μL of CCK-8 reagent (Dojindo), absorbance was measured at 450 nm using a microplate reader. For EdU staining, stably transfected cells were seeded at 5×10^4 cells per well in 24-well plates, allowed to adhere overnight, and then stained using the EdU kit (Beyotime) according to the manufacturer's instructions to evaluate proliferative capacity.

3.3 Wound Healing Assay

HCC cells (1×10^6 cells/well) were seeded in 6-well plates and cultured in DMEM without FBS for 24 hours. Wounds were created using a 200 μL pipette tip, and images were captured at 0 and 48 hours. The cell-free area was quantified to assess cell migration.

3.4 Transwell Assay

Invasion was assessed using 24-well transwell plates with 8 μm pore size, with or without Matrigel coating. Cells (1×10^5) were seeded in serum-free medium in the upper chamber, while 10% FBS medium was placed in the lower chamber. After 24–72 hours, cells in the upper chamber were removed, and invading cells were fixed with 4% PFA and stained with 0.1% crystal violet solution.

3.5 Cell Apoptosis Analysis

HCC cells were grown to 80% confluence in 6-well plates and then serum-starved by replacing the culture medium with FBS-free medium for 24 hours. Cells were harvested and apoptosis was detected using the Annexin V-FITC Apoptosis Detection Kit (Unitech). Data were analyzed using FlowJo software (v10.6.0).

3.6 Cell Cycle Analysis

For cell cycle synchronization, HCC cells were serum-starved (0.05% serum) for 48 hours to arrest them in the G0 phase, then reintroduced into medium with 10% FBS for cell cycle progression. Cell cycle distribution was analyzed using a Cytoflex flow cytometer (Beckman Coulter) according to the manufacturer's protocol for the cell cycle staining kit (Unitech).

3.7 Single-Cell RNA Sequencing and Analysis

After obtaining written informed consent from patients and their families, 4 pairs of HCC tissues and matched non-tumor tissues were collected for single-cell RNA sequencing. Cellranger v7.1.0 was used to align and quantify sequencing reads using the GRCh38 reference genome. Gene-barcodes matrices were analyzed using the Seurat R package (version 4.4.0), with doublets filtered using the DoubletFinder R package. All samples were merged into one Seurat object, and quality control was applied according to sample-specific standards. The "FindVariableFeatures" function identified the top 2000 most variable genes for principal component analysis (PCA). The "FindNeighbors" function was used for graph clustering based on PCA, and "FindCluster" was applied to obtain cell subtypes. t-SNE was used for visualization, and batch effects were corrected using the Harmony algorithm. Cells were categorized into hepatocytes, DC cells, T/NK cells, B cells, endothelial cells, epithelial cells, fibroblasts, macrophages, mast cells, and neutrophils.

3.8 Co-IP/MS

Huh7 cells were lysed in NP-40 buffer (50 mM Tris-HCl, pH 7.5, 150 mM NaCl, 0.5% NP-40, 1 mM PMSF) on ice. Cell extracts (1 mL) were incubated with 7 μg primary antibody for 2 hours, then with Protein A beads (10 μL) at 4°C for 45 minutes. After treatment with 2.5% SDC/100 mM Tris-HCl (pH 8.5) at 95°C, the supernatant was precipitated with TCA and resuspended in reduction/alkylation buffer (1% SDC, 100 mM Tris-HCl, 10 mM TCEP, 40 mM CAA), incubated at 60°C. Peptides were digested overnight with trypsin (1:50) and purified using SDB-RPS columns, then vacuum-dried and stored at -20°C. LC-MS/MS was performed using an UltiMate 3000 RSLCnano system and Q Exactive HF mass spectrometer. Peptides were separated on C18 columns using a gradient of mobile phases A (0.1% formic acid/3% DMSO/97% H_2O) and B (0.1% formic acid/3% DMSO/97% ACN) at 300 nL/min. MS was operated in DDA top20 mode (350-1500 m/z), with HCD fragmentation (28 NCE), 60,000 resolution at m/z 200, AGC target 3E6, and 30 ms max injection time. Fragmentation scans were at 15,000 resolution. Beads were washed with NP-40 buffer, immunocomplexes were eluted, separated by SDS-PAGE, and stained using a silver staining kit (Beyotime).

3.9 GST pulldown

Recombinant GST-tagged proteins were expressed in Escherichia coli, purified using Glutathione-Sepharose beads, and incubated with cell lysates containing potential interacting proteins. The mixture was rotated at 4°C for 2 hours to allow binding. After incubation, the beads were washed three times with cold PBS containing 0.1% NP-40 to remove non-specifically bound proteins. The bound proteins were eluted by boiling the beads in SDS-PAGE loading buffer and analyzed by Western blotting to detect interaction partners. Protein complexes were visualized using enhanced chemiluminescence.

3.10 Immunohistochemical (IHC) and immunofluorescence (IF) analysis

For IHC, paraffin-embedded tissue sections were dewaxed by heating at 65°C for 2 hours, followed by washing with PBS. Antigen retrieval was performed by microwaving sections in EDTA buffer, transitioning between moderate and boiling heat. After cooling, the sections were treated with 3% hydrogen peroxide, blocked with 5% BSA, and incubated overnight with the primary antibody at 4°C. After PBS washes, sections were incubated with secondary antibody for 50 minutes at 37°C and developed with DAB. Counterstaining with hematoxylin and dehydration

in alcohol and xylene was followed by mounting with neutral gum. For IF, cells were fixed with 4% paraformaldehyde, permeabilized with 0.5% NP-40, and blocked with 5% BSA. Cells were then incubated with primary and secondary antibodies, and cell nuclei were stained with DAPI. Images were acquired using a confocal microscope (SP8 AT CIAN, Leica).

3.11 Docking Analysis

The three-dimensional crystal structures of CCT2, ALDOA, and GSTP1 were obtained from the RCSB Protein Data Bank (PDB). Protein-protein docking analysis was conducted using the HDOCK server, which utilizes a hybrid algorithm integrating template-based modeling and free docking to predict interaction interfaces. The resulting protein-protein complexes were visualized using PyMOL, with key binding interfaces highlighted to illustrate molecular interactions.

3.12 Nanoparticle tracking analysis (NTA)

Nanoparticle tracking analysis was performed using a ZetaView Particle Metrix instrument (Particle Metrix, PMX-120, Germany) to evaluate the counting and size of exosomes. The instrument was calibrated using 100 nm polystyrene beads (Thermo Fisher Scientific) prior to use. After calibration, the NTA software helps quantify the nanoparticle concentration (particles/ml). To simplify the process, the batch function integrated in the NTA software was used for single sample measurements.

3.13 Atomic force microscopy (AFM)

To characterize the morphology of exosomes, samples were diluted in deionized water at a ratio of 1:1000 and then adsorbed onto freshly split mica sheets. After rinsing with deionized water, the samples were dried with a gentle stream of nitrogen. The surface morphology was then analyzed using an atomic force microscope (Shimadzu) in tapping mode. A silicon probe (OMCL-AC240TS-R3, OLYMPUS, resonant frequency 70 kHz, spring constant k~1.7 N/m) was used during the examination.

3.14 Transmission electron microscopy (TEM)

Exosomes were characterized by applying transmission electron microscopy. Purified exosomes suspended in PBS were dropped onto a copper grid. After staining with 2% uranyl acetate solution, the copper grids were allowed to dry under an infrared heat lamp and then observed by transmission electron microscopy (Thermo Fisher Scientific).

3.15 Cryo-transmission electron microscope (Cryo-TEM)

The exosomes were dissolved in 20 μL of PBS and placed on a glow discharge copper grid. Subsequently, the copper grids were wiped with a force of 3 for 3 seconds in a chamber with 100% humidity and 8°C. The prepared grids were then quickly immersed in liquid nitrogen pre-cooled liquid ethane using FEI's Vitrobot. Data sets were acquired using a Glacios 200 kV transmission electron microscope (Thermo Fisher Scientific) equipped with a Falcon 4 direct electron detector (Ceta D CMOS camera).

3.16 Labeling of Exosomes for Uptake Analysis

Exosomes were fluorescently labeled with DiR (DuoLaiMi) or PKH67 (MedChemExpress)

according to the manufacturer's protocol. The labeled exosomes were washed with PBS and collected by ultracentrifugation as described above. Cells were cultured with DiR-stained exosomes for 24 h. After 24 h of culture, cells were washed three times with PBS and then fixed with 4% paraformaldehyde (PFA) for 30 min at room temperature (RT). Cell nuclei were stained with DAPI (Thermo Fisher) for 15 min at room temperature. Images were acquired using a confocal microscope (SP8 AT CIAN, Leica).

3.17 Immuno-electron microscopy

Immuno-electron microscopy was used to assess the protein expression on exosomes. For immunogold labeling, isolated exosomes in PBS were dropped to glow-discharged copper grids, subjected to blocking, and incubated with a rabbit anti-human monoclonal antibody to recognize the CCT2 protein. Subsequently, the grids underwent incubation with an anti-rabbit secondary antibody, which was conjugated with colloidal gold particles. The sizes of the colloidal gold particles conjugated to the two secondary antibodies were 10 nm (Solarbio, K1034G-G10, CHN) and 4 nm (Jackson ImmunoResearch Laboratories Inc, 111-185-144, USA), respectively. Each staining step involved five PBS washes and ten ddH_2O washes before performing contrast staining with 2% uranyl acetate. Subsequently, the datasets were acquired using a transmission electron microscope (Thermo Fisher Scientific).

3.18 Enzyme-Linked Immunosorbent Assay (ELISA)

The expression levels of IL-10, TGF-β, and TNF-α were quantified using ELISA kits (Multi Sciences), following the manufacturer's instructions. All assays were performed in accordance with the standardized protocol provided in the kit to ensure accuracy and reproducibility.

3.19 Co-Culture Assay

Both Transwell co-culture and direct co-culture systems were employed to assess the interaction between hepatocellular carcinoma cells and macrophages. For Transwell co-culture, experiments were conducted using 24-well plates equipped with 0.4 μm pore-size inserts (Millipore, USA). THP-1 cell-derived M0 macrophages were seeded in the lower chamber at a density of 1×10^5 cells per well, while HCC cells (1×10^5 cells per well) were added to the upper chamber. After 48 hours, the culture media were collected for ELISA, and THP-1 cells were harvested for further analysis. For direct co-culture, exosomes isolated from HCC cells were directly added to macrophages at a final concentration of 1×10^{10} particles/mL.

3.20 RNA extraction and real-time quantitative PCR (qRT-PCR)

Total RNA was extracted with TRIzol (Takara). qRT-PCR was performed using HiScript Q RT Supermix (Vazyme) to reverse transcribe the total RNA. Total RNA was extracted with TRIzol and reverse transcribed using HiScript Q RT Supermix. qRT-PCR was performed using the 2ChamQ Universal SYBR qPCR Master Mix (Vazyme).

3.21 Western blot analysis

Total protein was extracted with RIPA (Solarbio). After electrophoresis, protein extracts were transferred to PVDF membranes and incubated with the corresponding antibodies. Color development was performed using ECL reagent (Bio-Rad) and Bio-Rad ChemiDoc XRS+.
